# Ultrafast Sulfur Redox Dynamics Enabled by a PPy@N-TiO_2_ Z-Scheme Heterojunction Photoelectrode for Photo-Assisted Lithium–Sulfur Batteries

**DOI:** 10.1007/s40820-025-01946-3

**Published:** 2026-01-01

**Authors:** Fei Zhao, Yibo He, Xuhong Li, Ke Yang, Shuo Chen, Yuanzhi Jiang, Xue-Sen Wang, Chunyuan Song, Xuqing Liu

**Affiliations:** 1 State Key Laboratory of Solidification Processing, Center of Advanced Lubrication and Seal Materials, School of Materials Science and Engineering, Northwestern Polytechnical University, Xi’an, 710072 People’s Republic of China; 2 Research and Development Institute of Northwestern, Polytechnical University in Shenzhen, Shenzhen, 518063 People’s Republic of China; 3 Key Laboratory of Advanced Energy Materials Chemistry (Ministry of Education), Nankai University, Tianjin, 300071 People’s Republic of China; 4 Department of Physics, National University of Singapore, Singapore, 117543 Singapore

**Keywords:** Photo-assisted lithium–sulfur batteries, Z-scheme heterojunction, Electrocatalysis, Photocatalysis, Sulfur redox dynamics

## Abstract

Photo-assisted lithium–sulfur batteries (PALSBs) offer an eco-friendly solution to address the issue of sluggish reaction kinetics of conventional LSBs. However, designing an efficient photoelectrode for practical implementation remains a significant challenge. Herein, we construct a free-standing polymer–inorganic hybrid photoelectrode with a direct Z-scheme heterostructure to develop high-efficiency PALSBs. Specifically, polypyrrole (PPy) is in situ vapor-phase polymerized on the surface of N-doped TiO_2_ nanorods supported on carbon cloth (N-TiO_2_/CC), thereby forming a well-defined p–n heterojunction. This architecture efficiently facilitates the carrier separation of photo-generated electron–hole pairs and significantly enhances carrier transport by creating a built-in electric field. Thus, the PPy@N-TiO_2_/CC can simultaneously act as a photocatalyst and an electrocatalyst to accelerate the reduction and evolution of sulfur, enabling ultrafast sulfur redox dynamics, as convincingly validated by both theoretical simulations and experimental results. Consequently, the PPy@N-TiO_2_/CC PALSB achieves a high discharge capacity of 1653 mAh g^−1^, reaching 98.7% of the theoretical value. Furthermore, 5 h of photo-charging without external voltage enables the PALSB to deliver a discharge capacity of 333 mAh g^−1^, achieving dual-mode energy harvesting capabilities. This work successfully integrates solar energy conversion and storage within a rechargeable battery system, providing a promising strategy for sustainable energy storage technologies.

**Supplementary Information:**

The online version contains supplementary material available at 10.1007/s40820-025-01946-3.

## Highlights

- A novel polymer–inorganic hybrid photoelectrode (PPy@N-TiO_2_/CC) with a Z-scheme heterostructure was first constructed for high-efficiency photo-assisted lithium–sulfur battery (PALSB).
- PPy@N-TiO_2_/CC acts not only as a photocatalyst to accelerate sulfur redox reductions through photocatalytic, photoconductive, and photo-charge effects, but also as an electrocatalyst to facilitate intermediate polysulfide conversion.
- PALSB achieves an ultrahigh discharge capacity of 1653 mAh g^−1^ and dual-mode energy harvesting: 5 h of photo-charging delivers a discharge capacity of 333 mAh g^−1^.

**Supplementary Information:**

The online version contains supplementary material available at 10.1007/s40820-025-01946-3.

## Graphical abstract

**Figure:**
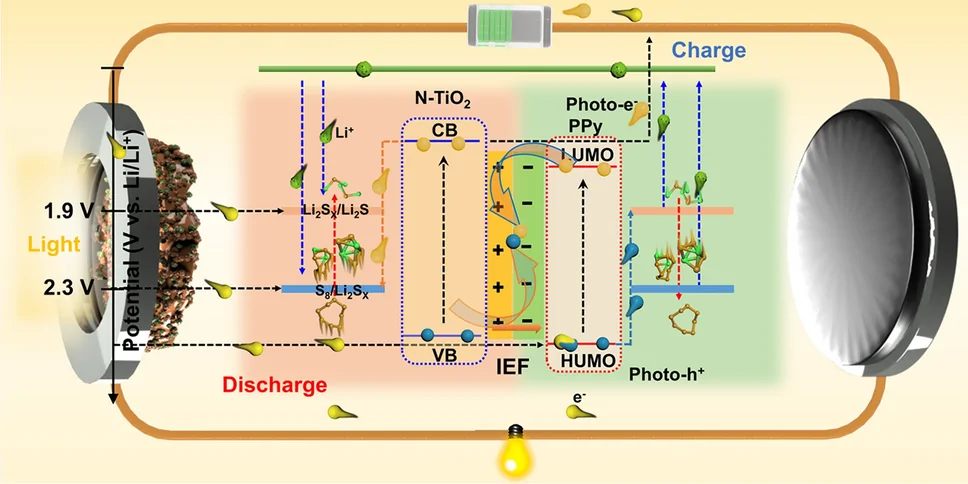

## Introduction

The depletion of resources and environmental pollution urge us to develop recyclable clean energy rapidly [1, 2]. Among various rechargeable batteries, the lithium–sulfur battery (LSB) is an ideal candidate for next-generation energy storage system owing to its high theoretical energy density (2600 Wh kg^−1^) [3–8]. However, there is still a significant gap between the actual performance of LSBs and the theoretical potential, primarily due to the sluggish/incomplete conversion of sulfur and its discharge intermediates, lithium polysulfides (Li_2_S_*x*_, 4 ≤ *x* ≤ 8) [9–11]. Fortunately, the conversion of polysulfide can be effectively promoted by introducing physical fields (such as optical fields, magnetic fields, and sound fields) [12]. Optical fields accelerate the liquid-to-solid conversion kinetics of polysulfides through photocatalytic effect and a magnetic field can optimize the mass transfer process of polysulfide through Lorentz force, while ultrasonic cavitation can significantly reduce the activation energy of polysulfide conversion [13–15]. Notably, the photo-assisted strategy can not only photoelectric co-catalyze the conversion of polysulfides, but also convert solar energy into chemical energy in a single device, exhibiting promising application prospects [16–18]. The design of an effective photocathode for capturing solar energy and storing it in LSBs allows photo-generated carriers to enhance the electrochemical reaction and reduce electric energy consumption, thereby facilitating the integration of solar energy storage and conversion within LSBs [19–21]. However, designing an effective photoelectrode remains a significant challenge.

Research on photo-assisted lithium–sulfur batteries (PALSBs**)** is still in its infancy. Up to now, only a few materials have been explored that qualified for constructing photoelectrodes for PALSBs. In 2015, a CdS/Pt photocathode was fabricated to assemble PALSB, which achieved a capacity of 792 mAh g^−1^ after 2 h light irradiation, confirming the great potential of integrating solar energy with LSBs [22]. Subsequently, CdS-TiO_2_ [23] and CdS/rGO [24] photoelectrodes were successively constructed to enhance the energy conversion efficiency and boost the electrochemical kinetics. Additionally, Ru-based dye (N719) was found to be effective in facilitating charge separation [25, 26]. Perovskite materials, commonly used in solar cells to prepare electrodes, were also shown to be capable of achieving solar-to-chemical energy conversion [27–29]. More recently, titanium-based metal–organic framework (Ti-MOF) bifunctional photocatalyst based on titanium-benzene-1,4-dicarboxylate (Ti-BPDC) with ligand defects was proposed, which exhibited excellent light harvesting capability, high electronic conductivity, low recombination rate of carriers, and electrochemical endurance [30]. In addition, photoelectrodes based on Bi_2_O_3_ [31] and Co_3_O_4_ [32] also showed superior catalytic ability for polysulfide conversion. These works proposed innovative strategies that can effectively enhance the comprehensive electrochemical performance, blazing the trail for the application of solar energy in LSBs. Inspired by these works, we developed a free-standing photoelectrode by constructing a heterostructured Au-loaded N-doped TiO_2_ on carbon cloths (Au@N-TiO_2_/CC), which accelerated the reduction and evolution of sulfur through the synergistic effect of electrocatalysis and photocatalysis [33]. However, it is regrettable that the photoelectronic conversion efficiency of photoelectrodes remains unsatisfactory due to the single type of semiconductor structure. More effective photoelectrodes with heterostructures for PALSB still need to be explored, and the relevant mechanism of photoelectric co-catalysis also should be elucidated.

Herein, a free-standing polypyrrole (PPy) modified N-TiO_2_/CC (PPy@N-TiO_2_/CC) photoelectrode was constructed and developed for PALSB. The p–n heterojunction formed between N-TiO_2_ and PPy facilitates the separation of photo-generated carriers by constructing a built-in electric field (IEF) to enhance the utilization efficiency of solar energy, which accelerates the redox kinetics of sulfur through the photocatalytic effect and the photoconductivity effect, which are systematically demonstrated by Raman spectra, in situ electrochemical impedance spectroscopy (EIS), and distribution of relaxation time (DRT) analysis. Consequently, the PPy@N-TiO_2_/CC assembled PALSB not only achieves an ultrahigh specific capacity with excellent rate performance, but also exhibits good cycling performance. Furthermore, the PALSB is capable of delivering a high discharge capacity during direct photo-charging while maintaining stability.

## Experimental Section

### Materials

Carbon cloths (WOS1011) were purchased from CeTech (Taiwan). Titanium butoxide (C_16_H_36_O_4_Ti, 99.0%), isopropanol (99.7%), acetone (99.5%), ethanol (99.7%), and hydrochloric acid (36–38%) were obtained from Sinopharm Chemical Reagent Co., Ltd. Pyrrole monomer (Py) (99%), ammonium persulfate (APS, ≥ 98%), and sodium dodecyl sulfate (SDS, 99%) were purchased from Beijing InnoChem Science & Technology Co., Ltd. (China). Melamine, tetraethylene glycol dimethyl ether, lithium sulfide, and sulfur powder were purchased from Shanghai Aladdin Bio-Chem Technology Co., Ltd. (China).

### Preparation of TiO_2_/CC, N-TiO_2_/CC and PPy@N-TiO_2_/CC

#### ***Preparation of TiO***_***2***_***/CC***

2.5 mL of titanium butoxide was added to 47.5 mL of isopropanol solution and stirred for 1 h. Commercial carbon cloths (CC) were immersed in the above solution for 10 min, followed by drying at 60 °C. The process of dipping-drying was repeated three times, and the CC were held in a tube furnace at 500 °C for 1 h. Subsequently, 1.32 mL of titanium butoxide was slowly added to 60 mL of a mixture of hydrochloric acid and deionized water (volume ratio of 1:1) and stirred for 6 h. The annealed samples with TiO_2_ crystalline species were immersed in the above mixed solution and hydrothermally treated at 150 °C for 12 h. Finally, the CC were rinsed with deionized water and dried for 12 h.

#### ***Preparation of N-TiO***_***2***_***/CC***

The N-TiO_2_/CC electrode was fabricated using the TiO_2_/CC electrode as a base. The TiO_2_/CC was put on a piece of carbon paper, then covered on a porcelain boat loaded with melamine powder, and heated up to 750 °C with an increase rate of 5 °C min^−1^, Finally, the samples were calcinated at 750 °C for 2 h in Argon gas atmosphere.

#### ***Preparation of PPy@N-TiO***_***2***_***/CC***

The prepared N-TiO_2_/CC was immersed in 20 mL of deionized water with 0.66 g APS and 0.01 g SDS for 30 min and subsequently transferred to a sealed glass tube containing 3 mL of pyrrole vapor. Polypyrrole was polymerized on its surface by exposure to pyrrole vapor for 20 min. The polymerization temperature was controlled at 25 °C. After polymerization, the samples were washed with ethanol and dried at room temperature (denoted as PPy@N-TiO_2_/CC). Finally, PPy@N-TiO_2_/CC was cut into electrodes with a diameter of 10 mm. PPy@CC was synthesized by replacing N-TiO_2_/CC with CC under the same conditions.

### Assembly of PALSB and Li_2_S_6_ Symmetric Battery

Different from the traditional LSB, the positive side of the PALSB was punched to collect sunlight, and the transparent window was sealed with epoxy resin glue. Commercial Celgard 2325 and Li metal were used as separators and anodes, respectively. The electrolyte was 1 M LiTFSI dissolved in DME and DOL (v/v = 1:1) with 2 wt% LiNO_3_. The sulfur loading was fixed at about 1 mg cm^−2^ added in the form of 0.5 M Li_2_S_6_ solution. The ratio between electrolyte volume and sulfur loading was 25. The assembly of Li_2_S_6_ symmetric battery is similar to the PALSB. The difference is that the counter electrode is a PPy@N-TiO_2_/CC electrode with 0.5 M Li_2_S_6_ solution. All batteries were assembled in an argon-filled glove box with moisture and oxygen levels below 0.01 ppm.

### Characterizations

X-ray diffraction patterns (XRD, D8 ADVANCE BRUKER, using Cu-K*α* radiation, *λ* = 1.5418 Å) were recorded to characterize the chemical compositions of the samples. The elemental composition and surface elemental state of samples were measured by X-ray photoelectron spectrometer (XPS, PHI 5000 VersaProbe III). Scanning electron microscope (SEM, Tescan Clara GMH) was used to analyze the morphologies of the materials. Chemical bonds of materials were investigated by Fourier transform infrared spectroscopy (FT-IR, Tensor II BRUKER Vertex 70). Photoluminescence emission spectra (PL, Hitachi-F4600) were measured to analyze the fate of photo-generated electron–hole pairs. The absorption spectra of the materials were characterized by UV–visible spectrophotometer (UV–Vis, Agilent Cary 7000). The relationship between optical band gap and absorption coefficient is as follows:

1 $$\left( {\alpha h\nu } \right)^{1/n} = A \, \left( {h\nu - E_{{\mathrm{g}}} } \right)$$

where *α* is the absorption coefficient, *h* presents the Planck constant, *ν* stands for the frequency of photo, *n* is related to the type of the semiconductor, *B* is constant, and *E*_g_ is the bandgap of the semiconductor.

### Electrochemical Measurements

A 300 W Xe lamp was used as solar simulator with a 350–780 nm filter, and the power density was fixed at 60 mW cm^−2^. The cyclic voltammetry (CV) test voltage window of the LSB and the symmetrical cell is 1.7–2.8 V and −0.6 to 0.6 V, respectively. The frequency of the electrochemical impedance spectra (EIS) test is 10^5^ to 0.01 Hz. The above tests are all evaluated on the CHI 660E electrochemical workstations. The galvanostatic charge/discharge (GCD) and cycling performance of the electrodes were evaluated on Neware BTS 7.5 × battery test system. Mott–Schottky (M–S) plots were measured according to a three-electrode system in 0.5 M Na_2_SO_4_ solution (pH = 7.0), with a Pt foil counter-electrode and Ag/AgCl reference electrode.

The diffusion coefficient of Li^+^:

2 $$Ip = 2.69 \times 10^{5} \cdot n^{3/2} \cdot S \cdot D_{{{\mathrm{Li}}}}^{1/2} \cdot C_{{{\mathrm{Li}}}} \cdot v^{1/2}$$

where *Ip* is the peak current, n represents the number of transferred electrons (*n* = 2 in LSB), *S* stands for the area of the test electrode, *D*_Li_ corresponds to the diffusion coefficient of Li^+^, *v* denotes the scanning rate, and *C*_Li_ refers to the concentration of Li^+^ involved in redox in the system.

The overall solar energy conversion efficiency (*η*_SCE_):

3 $$\eta_{{{\mathrm{SCE}}}} = E_{{{\mathrm{out}}}} /\left( {P_{{{\mathrm{in}}}} \times t \times S} \right) \times 100\%$$

where *E*_out_ is the discharge energy, *P*_in_ stands for the light power density, *t* refers to the photo-charging time, and *S* represents the active photocathode area. Specifically, the transparent window of the PALSB is 8 mm, and the actual illuminated area is approximately 0.5 cm^2^. During the photoelectric charging process, the xenon lamp power is fixed at about 60 mW cm^−2^.

### Details of the Theoretical Simulations

The calculations in this study were performed by applying density functional theory (DFT) methods implemented in the Vienna ab initio simulation package (VASP). The frozen core all-electron projector-augmented-wave (PAW) method was used to describe the interactions between electrons and other particles. The exchange–correlation effect was considered by the standard generalized gradient approximation (GGA) under the Perdew–Burke–Ernzerhof (PBE) scheme. The cutoff energy for plane-wave basis was set to 400 eV. A Monkhorst–Pack k-mesh of 3 × 2 × 1 was used to sample the first Brillouin zone. The semiempirical DFT-D3 dispersion correction with Becke–Johnson damping is employed to elucidate the weak van der Waals (VDW) interaction. The convergence threshold for energy and force was set to 10^−6^ eV and 0.01 eV Å^−1^, respectively.

## Results and Discussion

### Design Principal of PPy@N-TiO_2_/CC Photoelectrode

**Fig. 1:**
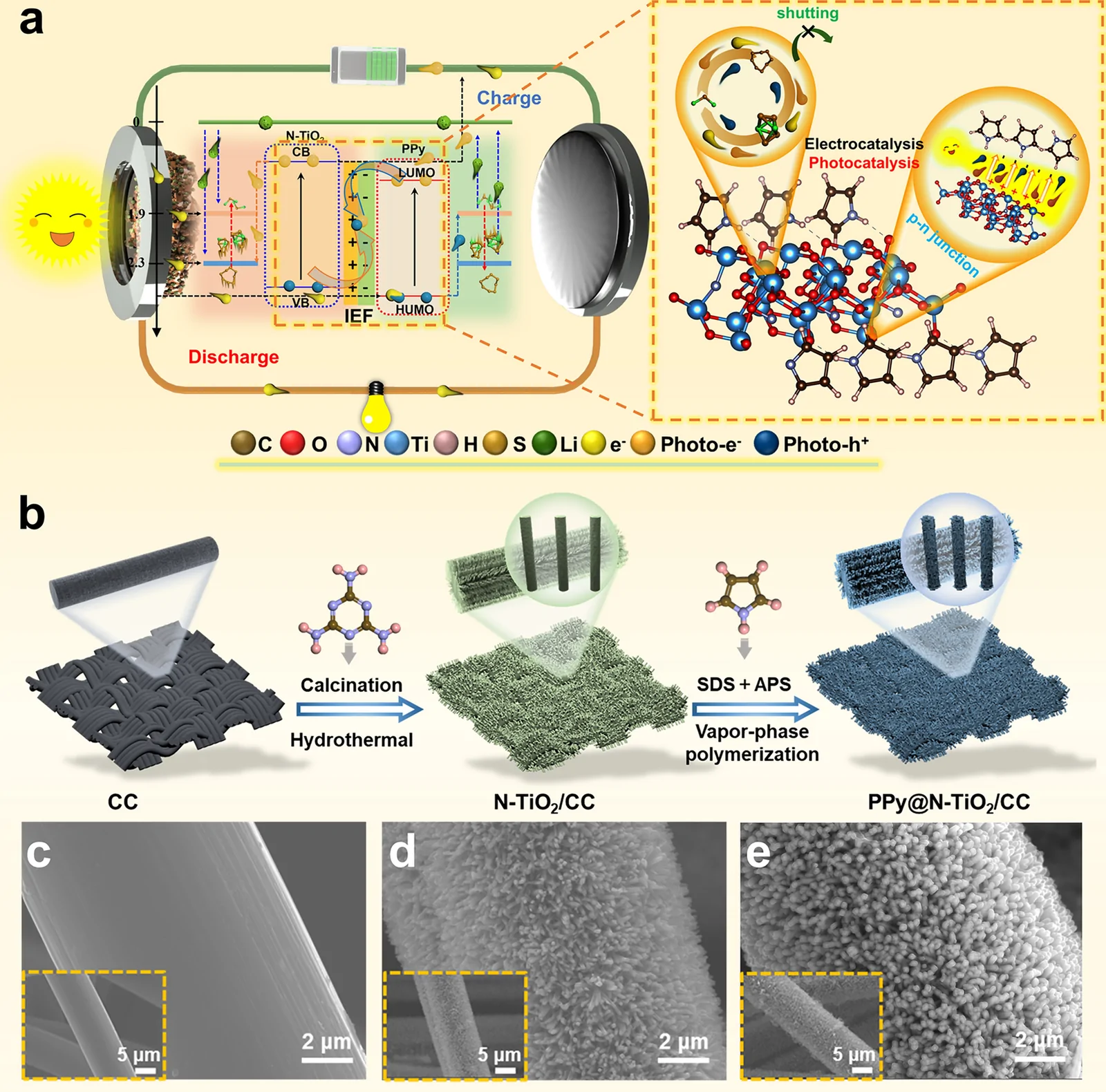
**a** Schematic diagram illustrating the working mechanism of PALSB with a PPy@N-TiO_2_/CC photoelectrode. **b** Schematic illustrating the fabrication progress of PPy@N-TiO_2_/CC. SEM images of **c** CC; **d** N-TiO_2_/CC and **e** PPy@N-TiO_2_/CC

Figure 1a presents a schematic diagram illustrating the design principle and working mechanism of a PALSB with a PPy@N-TiO_2_/CC photoelectrode. It is well-established that TiO_2_ is a typical semiconductor with excellent optical properties and stability, but its light utilization and photocatalytic efficiency are limited by the wide band gap and high carrier recombination rate [34–36]. Herein, N-doping is employed to narrow the band gap of TiO_2_ and improve the visible-light catalytic activity. Furthermore, p-type polymer semiconductor PPy with distinctive electrical and optical properties is introduced to enhance the carrier separation rate and improve the visible-light absorption coefficient by forming p–n heterostructure. To be specific, the Fermi level (*E*_f_) of n-type semiconductor N-TiO_2_ is close to the conduction band (CB) while the *E*_f_ of p-type semiconductor PPy is close to the highest occupied molecular orbital (HOMO) position. When they combine to form a p–n heterojunction, the carriers are redistributed to level the *E*_f_. As a result, the IEF from N-TiO_2_ to PPy is successfully established within the direct Z-scheme heterojunction PPy@N-TiO_2_/CC, thus improving the carrier separation rate and prolonging the lifetime of photoelectrons/holes. More specifically, under light illumination, photoelectrons are excited from the valence band (VB)/HOMO to the conduction band (CB)/lowest unoccupied molecular orbital (LUMO), while holes remain in the VB/HOMO. The photo-generated electrons and holes are effectively separated under the action of the IEF. During the discharge process, the photo-generated electrons facilitate the reduction of sulfur to polysulfides and further to insoluble Li_2_S. Meanwhile, the photo-generated holes are neutralized by electrons in the external circuit. During the charge process, the holes gradually oxidize Li_2_S to S_8_, effectively promoting the sulfur evolution reaction. Simultaneously, the photo-generated electrons reach the anode through the external circuit to reduce Li^+^ to Li, which is the reverse reaction of the discharge process. Consequently, the synergistic effect of photocatalysis and electrocatalysis accelerates the bidirectional conversion of sulfur during battery operation, thereby enhancing the utilization of active material and relieving the shuttle of polysulfides.

Figure 1b illustrates the fabrication progress of the free-standing PPy@N-TiO_2_/CC photoelectrode. Firstly, dense TiO_2_ nanorods are uniformly grown in situ on the carbon cloths through a hydrothermal method. Subsequently, nitrogen-doped TiO_2_ is prepared via a thermal treatment of melamine. Finally, PPy is vapor-phase polymerized on the surface of N-TiO_2_ nanorods to obtain PPy@N-TiO_2_/CC photoelectrode. The microstructure and morphology of the products at each stage were observed. As shown in Figs. 1c and S1, the CC are woven from carbon fibers with a diameter of about 10 μm. After the hydrothermal process, TiO_2_ nanorods grow uniformly and vertically on the surface of carbon fiber (Fig. S2a, b). The doping of N element did not change the rod-like structure of TiO_2_ (Fig. 1d). The vapor-phase polymerized PPy is compactly and uniformly coated on the surface of the N-TiO_2_ nanorods (Figs. 1e and S3), providing abundant active sites to facilitate the sulfur redox reaction. As depicted in Fig. S4, the elemental mapping demonstrates the uniform distribution of Ti, O, and N elements on the PPy@N-TiO_2_/CC, confirming the successful construction of the PPy@N-TiO_2_/CC heterostructure. Additionally, the ordered structure of PPy@N-TiO_2_/CC photoelectrode can provide fast-transferring electron channels and alleviate the volume expansion effect during the charge and discharge processes.

### Structure of PPy@N-TiO_2_/CC Photoelectrode

**Fig. 2:**
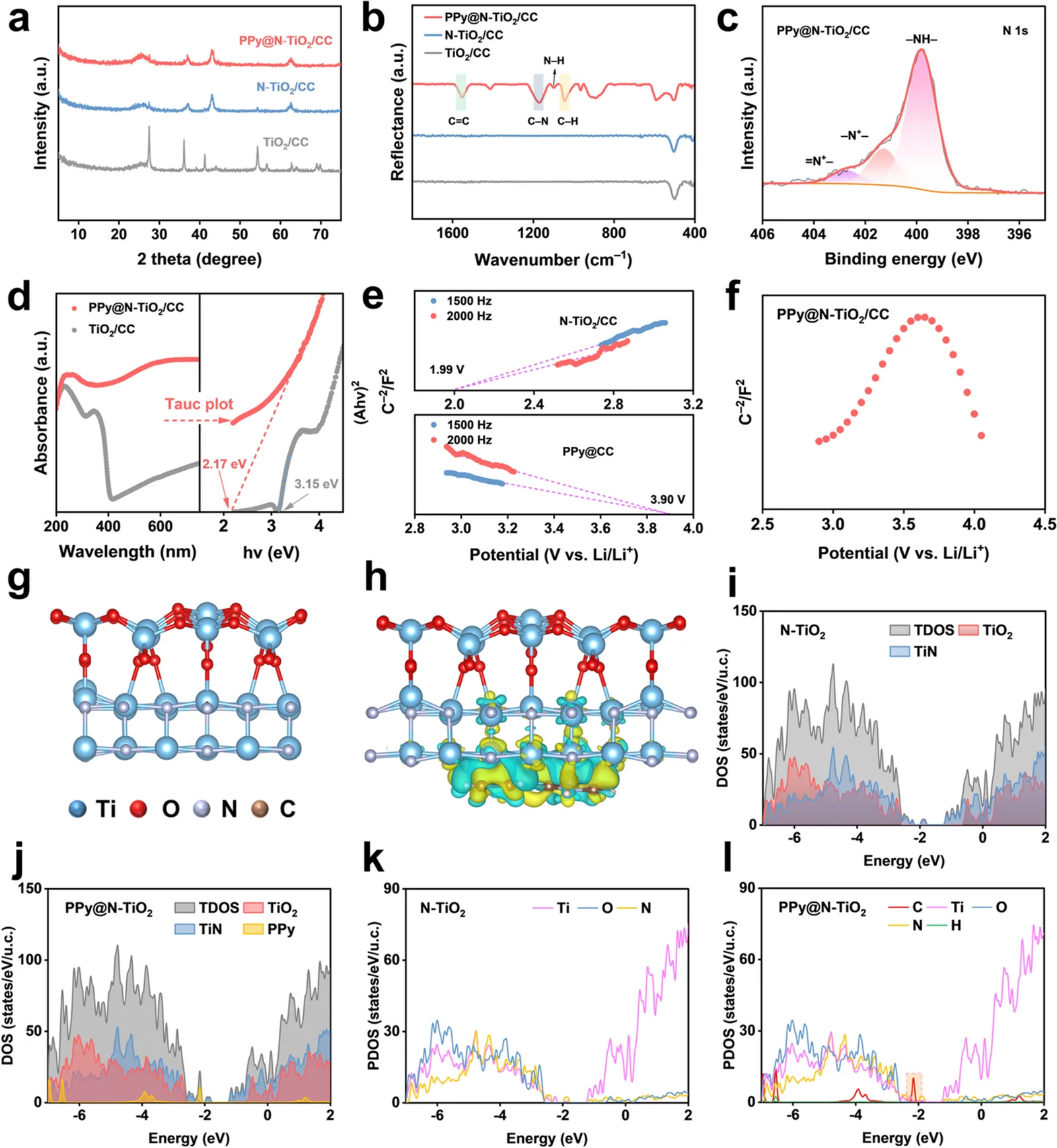
**a** XRD patterns and **b** FT-IR spectra of various samples. **c** N 1* s* high-resolution spectrum of PPy@N-TiO_2_/CC. **d** UV–Vis absorption spectra and Tauc plots of PPy@N-TiO_2_/CC and TiO_2_/CC. M-S plots of **e** N-TiO_2_, PPy@N-TiO_2_ and **f** PPy@N-TiO_2_/CC. DFT computational configuration of **g** N-TiO_2_ and **h** PPy@N-TiO_2_. TDOS plots for **i** N-TiO_2_ and **j** PPy@N-TiO_2_. PDOS plots for **k** N-TiO_2_ and **l** PPy@N-TiO_2_

The crystal structure of various samples was analyzed using X-ray diffraction (XRD) (Fig. 2a). TiO_2_ prepared by the hydrothermal method exhibits a pure rutile phase. The appearance of TiN characteristic peaks implies the successful introduction of N element. No corresponding characteristic peaks can be observed after vapor-phase polymerization of PPy, which may be attributed to the amorphous nature of PPy structure [37]. Fourier transform infrared (FT-IR) spectroscopy was employed to confirm the successful synthesis of PPy (Fig. 2b). The characteristic peak of PPy@N-TiO_2_/CC at 1551 cm^−1^ is assigned to the stretching vibration of the C=C bond on the pyrrole ring [38], whereas the peaks observed at 1165 and 1098 cm^−1^ correspond to the tensile vibrations of the C–N bond and N–H bond, respectively [39, 40]. Furthermore, the frequency at 1041 cm^−1^ is ascribed to the deformation of the in-plane C–H bond [41]. The chemical environment of PPy@N-TiO_2_/CC was further investigated with X-ray photoelectron spectroscopy (XPS). The high-resolution N 1*s* spectrum of PPy@N-TiO_2_/CC is shown in Fig. 2c. The peak at 399.8 eV corresponds to the –NH– group (neutral state) of pyrrole ring. The peaks at 401.2 and 402.9 eV are attributed to –N^+^– (polaron state) and =N^+^– (bipolaron state) in PPy, respectively. It is worth noting that PPy exhibits excellent electrochemical properties with its components in the oxidized state [42]. N-TiO_2_/CC shows a different high-resolution N 1*s* spectrum with PPy@N-TiO_2_/CC (Fig. S5), and it presents three peaks at 397.0, 398.7, and 400.8 eV, corresponding to Ti–N, Ti–N–O, and pyrrolic–N, respectively [43]. The C 1*s* spectrum of PPy@N-TiO_2_/CC further confirms that PPy has both polaron and bipolaron states (Fig. S6a), and the weak peak at 292.1 eV can be related to the π–π* satellite peaks of the aromatic structures [44–46]. Additionally, the peaks at 464.9, 463.0, 458.8, and 455.7 eV core levels in the Ti 2*p* XPS spectrum can be assigned to Ti^4+^ 2*p*_1/2_, Ti^3+^ 2*p*_1/2_, Ti^4+^ 2*p*_3/2_, and Ti^3+^ 2*p*_3/2_, respectively (Fig. S6b), confirming the formation of surface defect in PPy@N-TiO_2_ [47].

The light absorption performance of various photoelectrodes was evaluated by UV–visible (UV–Vis) absorption spectroscopy (Fig. 2d). TiO_2_/CC exhibited significant absorption only in the UV region, while PPy@N-TiO_2_/CC showed significant visible-light absorption, which is extremely important in determining the photocatalytic activity of the photoelectrode. According to Tauc plots, the band gap of PPy@N-TiO_2_/CC is calculated to be 2.17 eV, which is narrower than that of TiO_2_/CC (3.15 eV), implying that the incorporation of PPy and N atom effectively enhances the light absorption ability. Besides, the bandgaps of the N-TiO_2_/CC and PPy@CC were estimated to be 2.19 and 2.13 eV, respectively (Fig. S7a, b). Mott–Schottky (M-S) plots were recorded to determine the flat band potential (*E*_FB_) and the semiconducting properties of the heterojunctions. The N-TiO_2_/CC showed positive slopes, characteristic of an n-type semiconductor where electrons are majority carriers, while the PPy@CC is a p-type semiconductor with negative slopes (Fig. 2e). The presence of both positive and negative slopes in the M–S plot (inverted V-shape) is the evidence of the successful construction of a p–n heterojunction in PPy@N-TiO_2_/CC (Fig. 2f) [48]. Since the E_FB_ is 0.2 V more negative than the VB of p-type semiconductor or 0.2 V more positive than the CB of n-type semiconductor, it can be estimated that the CB/VB of N-TiO_2_/CC and PPy@CC were 1.79/3.98 and 1.97/4.10 V (vs. Li/Li^+^), respectively (Fig. S8). Photo-generated electrons and holes in semiconductors can recombine under photoexcitation conditions, so the carrier lifetimes were evaluated by photoluminescence (PL) spectroscopy. In comparison with N-TiO_2_/CC and TiO_2_/CC, PPy@N-TiO_2_/CC exhibited the lowest emission intensity (Fig. S9), suggesting that the formation of a p–n junction effectively suppresses the recombination of photo-generated electron–hole pairs and further improves the utilization of light energy.

The charge distribution of the PPy@N-TiO_2_ photocathode was analyzed by density functional theory (DFT) calculations. The optimized structural models of N-TiO_2_ and PPy@N-TiO_2_ heterostructures are shown in Fig. 2g, h. The configuration model remains stable prior to contact, and the yellow and blue areas represent the charge accumulation and charge depletion, respectively. It can be seen that there exists an obvious charge exchange effect between PPy and N-TiO_2_ interfaces, which effectively regulates the photo-generated carrier transport pathway, thus improving the catalytic activity. To reveal electron density difference between PPy and N-TiO_2_, the corresponding charge equilibrium plot is depicted at Fig. S10. It can be seen that electrons migrated from N-TiO_2_ to PPy, indicating the formation of a built-in electric field from N-TiO_2_ to PPy. To investigate the occupied states of the energy bands, the total density of states (TDOS) and partial density of states (PDOS) of the samples were calculated. The N-TiO_2_ and PPy@N-TiO_2_ electrodes exhibit excellent conductivity, which could be attributed to the successful introduction of N atoms (Fig. 2i, j). The PDOS analysis provides insight into the contribution of the valence orbitals from constituent atoms to charge migration with the composites. Figure 2k reveals that the CB and VB of N-TiO_2_ comprise contribution from Ti and N. For the PPy@N-TiO_2_, a new band emerges due to the orbital interaction of C, H of PPy and N of N-TiO_2_ near the VB, confirming the further enhancement of charge transfer capability within the composite (Fig. 2l). These results indicate that the synergistic interaction between PPy and N-TiO_2_ nanorods can facilitate the carrier transport rate, thereby improving the photocatalytic activity [49].

### Catalytic Mechanism of the PPy@N-TiO_2_/CC Photoelectrode

**Fig. 3:**
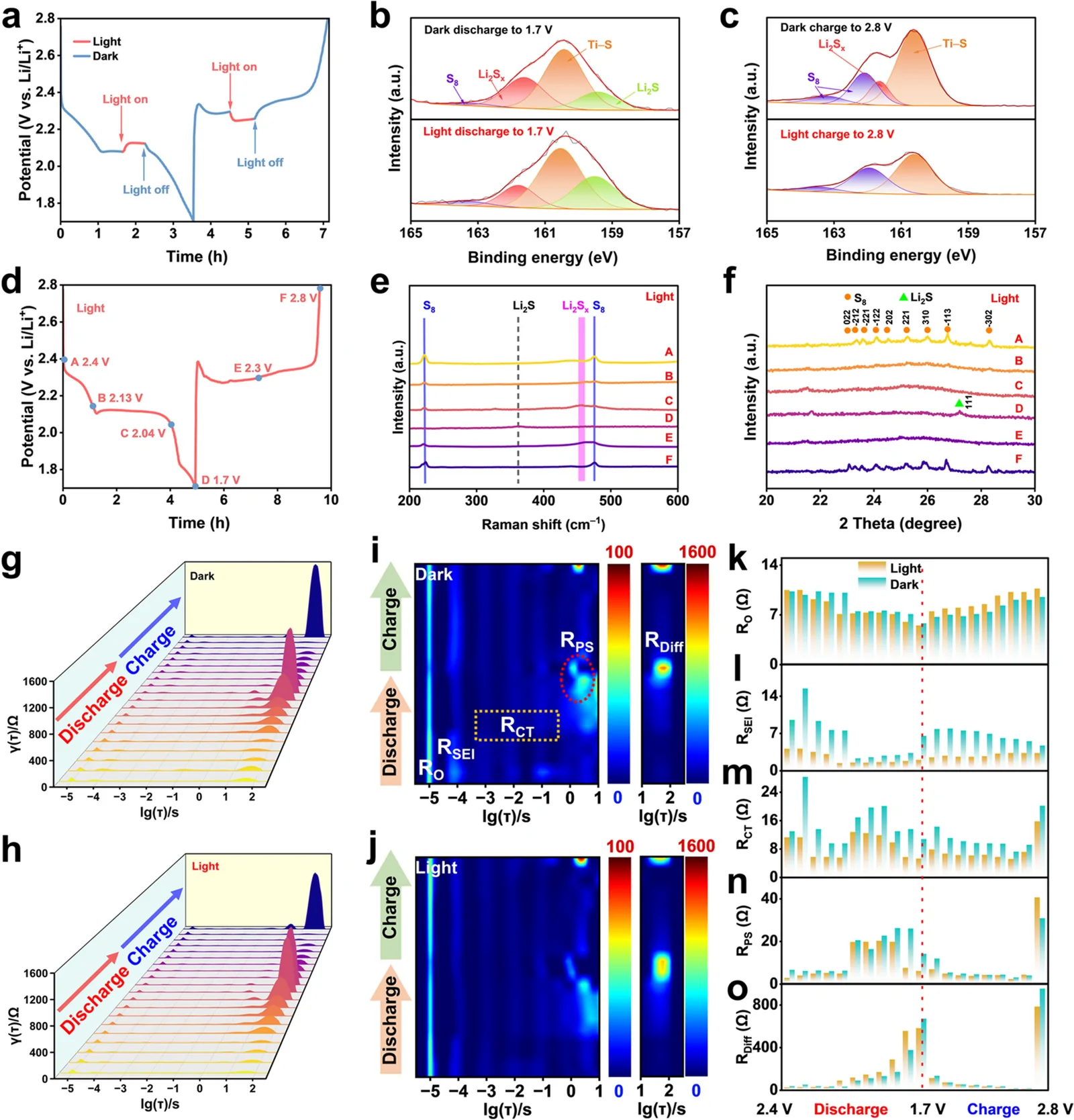
**a** GCD curves of PPy@N-TiO_2_/CC battery at 0.2 C by altering light on/off. S 2p XPS spectra of PPy@N-TiO_2_/CC photocathode at **b** 1.7 V and **c** 2.8 V. **d** GCD curves of PPy@N-TiO_2_/CC battery at 0.2 C. Corresponding **e** Raman spectra and** f** XRD patterns of PPy@N-TiO_2_/CC photocathode with the illumination. DRT calculated from in situ EIS measurements at different state-of-charge **g** without and **h** with the illumination. Corresponding 2D intensity color maps of the DRT calculated from EIS **i** without and **j** with the illumination. **k–o** Resistance contribution extracted from DRT profiles at 0.2 C

To investigate the impact of the photo-generated carriers on the electrochemical potential, the galvanostatic charge/discharge (GCD) curves of PALSB at 0.2 C were recorded by controlling the light on/off. As shown in Fig. 3a, the discharge voltage platform increased by 44 mV and the charge voltage platform decreased by 40 mV rapidly when light on. It can be noted that the charge/discharge capacity was also largely increased when the light was introduced (Fig. S11a, b). These results indicate that the photo-assisted effect effectively reduces the polarization of the battery, thereby enhancing the electrochemical reaction kinetics. XPS was employed to analyze the PPy@N-TiO_2_/CC electrode in both fully discharged and charged states in different lighting conditions. The binding energies at 159.6, 160.6, 162.1, and 163.5 eV correspond to Li_2_S, Ti–S bond, S_8_, respectively, while the peak at 161.6 eV is assigned to Li_2_S_*x*_. The relative area of Li_2_S increases from 14.32% (Dark) to 29.53% (Light) after discharging (Fig. 3b), and the similar results can be observed for S_8_ in fully charged state (from 26.59 to 43.14%) (Fig. 3c). Notably, it can be seen that the characteristic peak of Li_2_S_*x*_ almost disappeared when the battery is charged to 2.8 V under light irradiation. These results demonstrate that photo-generated carriers can effectively accelerate the polysulfides conversion, greatly enhancing the utilization of active species. The relevant charge/discharge mechanism of the PALSB was further conducted by Raman spectroscopy and XRD analysis. During discharging and charging processes, six characteristic voltage points were selected to study the process of the sulfur redox reduction with and without the illumination (Figs. 3d and S12a). As shown in Figs. 3e and S12b, the Raman characteristic peaks at 220 and 475 cm^−1^ correspond to S_8_. The intensity of S_8_ fades away gradually during the discharge process, and there are no obvious characteristic peaks of S_8_ in the fully discharged state, whereas the characteristic peak at 363 cm^−1^ ascribed to Li_2_S is observed, which is more obvious under the illumination. During the charge process, the characteristic peak of Li_2_S gradually disappears, and the characteristic peak signals of S_8_ reappear in the fully charged state. The polysulfide signal can be detected at 458 cm^−1^, and the intensity of the polysulfides signal is obviously weaker under light condition compared to dark condition throughout the whole reaction. Even in the fully charged state, a distinct polysulfide signal persists under dark conditions, which is consistent with the results of XPS analysis. The same tendency can also be detected by XRD test. As shown in Fig. 3f, the diffraction peaks at 23.07°, 23.38°, 23.67°, 24.01°, 24.51°, 25.12°, 25.91°, 26.65°, and 28.23° correspond to the (022), (−212), (−221), (−122), (202), (221), (310), (−113), and (−302) planes of α-S_8_, respectively. The characteristic peak of S_8_ progressively disappears, while the characteristic peak of Li_2_S (plane of 111) gradually emerges during discharging with the illumination. During the charge process, the diffraction peaks corresponding to S_8_ reappear. In comparison, the characteristic peaks of Li_2_S during discharge and S_8_ during charge are more ambiguous under dark conditions (Fig. S12c), indicating sluggish conversion kinetics of polysulfides, which further suggests that the introduction of photocatalyst into the LSBs exhibits significant superiority.

The DRT analysis of in situ EIS spectra was performed to further investigate the photo-assisted electrochemical process of LSBs. Figure S13a, b shows the Nyquist diagrams of the PALSBs with PPy@N-TiO_2_/CC photoelectrodes operated at 0.2 C with and without the illumination. The corresponding DRT fitting results are presented (Fig. 3g, h), and the 2D intensity color maps derived from the DRT analysis are shown in Fig. 3i, j. Figure S14 presents the electrochemical processes associated with distinct time constants. The peaks at 10^−5^, 10^−4^, 10^−3^–10^−1^, 10^−1^–10^1^, and 10^1^–10^2^ s correspond to the distributed ohmic resistance (*R*_O_), the migration of Li^+^ through the solid electrolyte interphase (SEI) impedance (*R*_SEI_), the charge transfer impedance of the positive electrode (*R*_CT_), the polysulfide diffusion impedance (*R*_PS_), and the ion diffusion impedance (*R*_Diff_), respectively [50]. During the discharging process, *R*_O_ is smaller under illumination than in the dark (Fig. 3k), which can be attributed to the photoconductivity effect. Conversely, during the charging process, *R*_O_ is much smaller in the dark, which is associated with the loss of active material as the dissolved polysulfides exposing more electrode area, indicating inferior electrochemical reversibility. The stability of the SEI is closely related to the electrochemical performance of LSBs [51]. There is a more obvious fluctuation of R_SEI_ in the dark compared to under illumination, implying a more stable electrochemical process in the presence of light (Fig. 3l). Specifically, the polysulfides are unable to complete the conversion in time under dark conditions. Consequently, they diffuse to the negative area and react with the lithium metal, causing the breakdown and collapse of the SEI film. The lower *R*_CT_ under illumination is attributed to the photoconductive effect (Fig. 3m), whereas the reduced *R*_PS_ results from the photocatalytic effect (Fig. 3n). It is noteworthy that the *R*_PS_ under illumination drops suddenly at the end of the discharge process, proving the rapid and complete conversion of polysulfides under light conditions. Meanwhile, the higher *R*_PS_ in the fully charged state also indicates that more polysulfides have been converted to S_8_. Additionally, the rapid increase in *R*_Diff_ under illumination further implies accelerated electrochemical reaction kinetics (Fig. 3o), which shortens the existence time of polysulfides and suppresses the side reaction.

### Electrochemical Performance of PALSBs

PALSBs were assembled with PPy@N-TiO_2_/CC photoelectrodes to investigate the effect of illumination on electrochemical performance. The schematic diagram and optical photograph of PALSB assembly are shown in Fig. S15. Different from conventional LSBs, PALSBs feature an 8 mm optical window on the positive case for solar energy harvesting, while employing PET membrane for encapsulation to maintain electrolyte system stability. To eliminate the thermal effect generated by infrared light, a 300 W Xe lamp equipped with a 350–780 nm filter was employed as a simulated light source. To explore the stability of the electrolyte of PALSB systems, Li||PPy@N-TiO_2_/CC batteries were assembled and subjected to linear sweep voltammetry (LSV) tests under light conditions. As shown in Fig. S16, the electrolyte system remained stable within the 1.7–2.8 V, while an obvious degradation onset was observed around 3.55 V. These results demonstrate that the introduction of light illumination can maintain electrolyte stability in LSB systems.

**Fig. 4:**
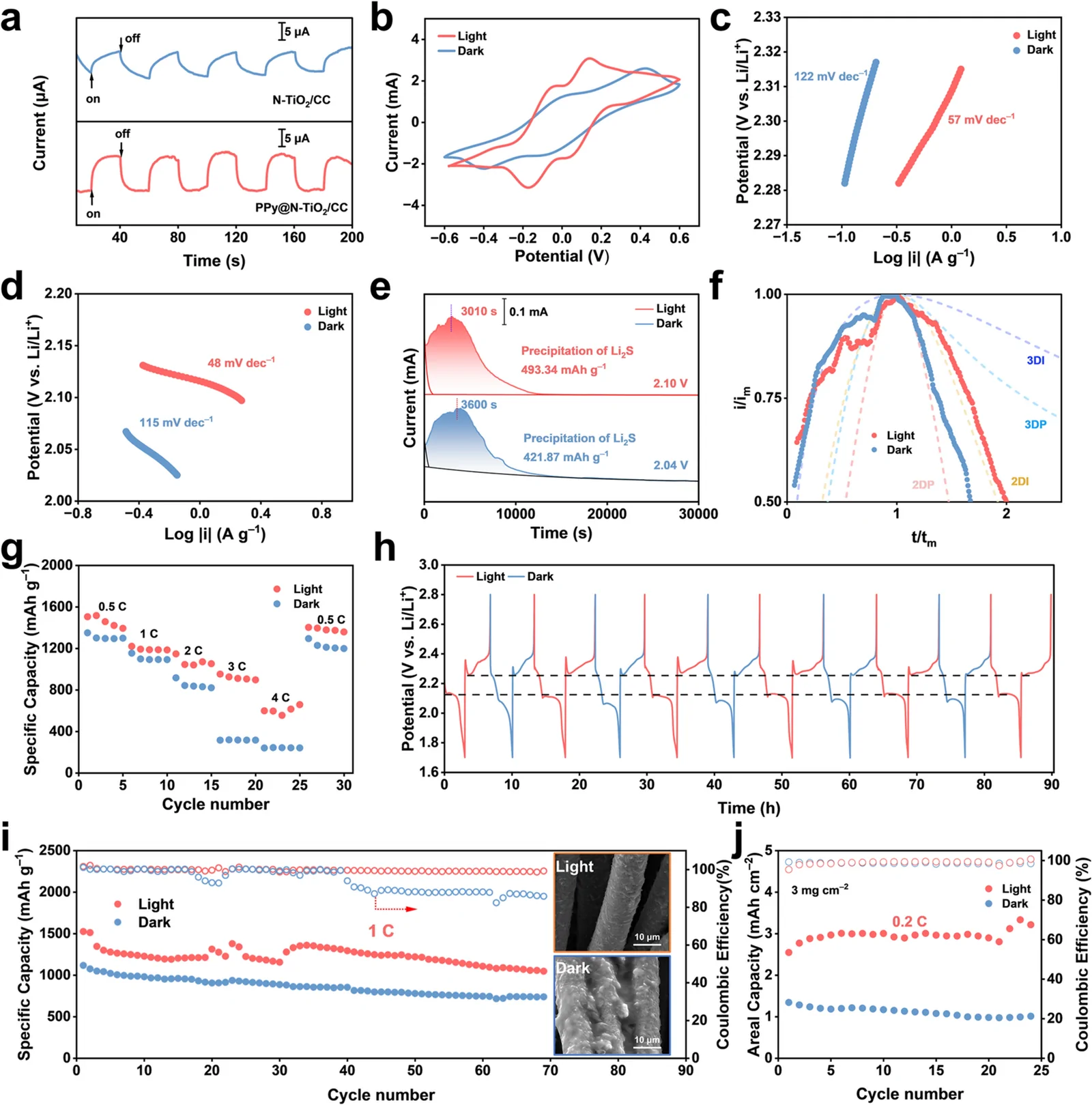
**a** Transient photocurrent response. **b** CV curves of Li_2_S_6_ symmetric batteries at 0.5 mV s^−1^ with a PPy@N-TiO_2_/CC electrode. **c, d** Tafel curves and **e** potentiostatic discharge curves of PPy@N-TiO_2_/CC battery. **f** Dimensionless current–time transient for the Li_2_S nucleation process of the PPy@N-TiO_2_/CC battery. Electrochemical performance of PPy@N-TiO_2_/CC photoelectrode with and without the illumination: **g** Rate performance; **h** charge/discharge potential curves by altering the illumination periodically at 0.2 C; **i** cycling performance at 1 C (inset: the SEM images of PPy@N-TiO_2_/CC electrodes after 69 cycles with and without the illumination); **j** cycling performance

As shown in Fig. 4a, the corresponding photocurrent signal appears for N-TiO_2_/CC, while the PPy@N-TiO_2_/CC possesses superior photo-response ability, confirming that the p–n heterojunction effectively reduces the recombination rate of carriers. Cyclic voltammograms (CV) curves of Li_2_S_6_ symmetric batteries at 0.5 mV s^−1^ using PPy@N-TiO_2_/CC electrode were obtained to evaluate the redox kinetics of polysulfide conversion under light illumination (Fig. 4b). There are two reversible redox peaks with or without the illumination, and the peaks appear earlier as well as larger response current under light irradiation, confirming the excellent electrocatalytic and photocatalytic performance of PPy@N-TiO_2_/CC. Simultaneously, the strong polysulfide affinity of PPy@N-TiO_2_/CC facilitates its catalytic role effectively (Fig. S17). It should be noted that there is no apparent response current in the ether-based electrolyte without Li_2_S_6_, indicating that the capacitive behavior of PPy@N-TiO_2_/CC is negligible (Fig. S18). The EIS spectra of the PPy@N-TiO_2_/CC assembled battery are shown in Fig. S19. In the equivalent circuits, R_1_ represents the ohmic internal resistance of the battery; the two semicircles in the high- and mid-frequency regions of the Nyquist plot correspond to the deposition of lithium polysulfides on the surface of the sulfur cathode (*R*_2_) and the charge transfer resistance (*R*_3_), respectively, while the straight line in the low frequency region is the Warburg impedance (*W*_1_) [52]. Compared to the non-illumination condition, the PALSB exhibits smaller R_1_ (6.4 Ω), R_2_ (12.5 Ω), and *R*_3_ (11.4 Ω). The electrochemical impedance tests under photothermal conditions were performed by sealing the light-transmitting window of PALSB by aluminum foil. As depicted in Fig. S20, compared with dark conditions, the reduction of impedance under photothermal conditions is limited, which indicates that the main reason for the improvement of the dynamic performance of charge transport kinetics of the system is caused by the photoconductivity effect (Table S1).

CV curves at various scan rates were obtained to evaluate the effect of illumination on the catalytic performance. As shown in Fig. S21a–d, the two reduction peaks in the CV curves with or without illumination correspond to the transformation of S_8_ to polysulfide intermediates and subsequent reduction to Li_2_S_2_/Li_2_S, respectively. With the illumination, the PALSB has an enhanced faradaic current, and the reduction peak exhibits higher initial/peak voltage, suggesting that photo-generated carriers accelerate the conversion of polysulfides and significantly reduce the reaction energy barrier of the liquid–solid conversion process. Meanwhile, the negative shift of oxidation peak and the increased response current indicate that the PALSB exhibits reduced polarization voltage and accelerated reaction kinetics. The positive effect of photocatalysis is further verified by Tafel fitting of anode peak and cathode peak according to the CV curves at 0.1 mV s^−1^. It can be observed that the Tafel slopes under illumination (57/48 mV dec^−1^) are significantly lower than those without the illumination (122/115 mV dec^−1^) (Fig. 4c, d). The smaller Tafel slopes further demonstrate the superior catalytic performance under illumination [53]. The influence of photoconductivity on Li^+^ diffusion in the PPy@N-TiO_2_/CC battery was investigated through CV test at different scan rates (Fig. S22a, b). The linear fitted curves of the oxidation and reduction peak currents versus the square root of the scan rate are shown in Fig. S22c, d. The fitting slope of PALSB is obviously higher than that under the unilluminated condition, indicating that photoconductivity effect increases the carrier concentration of the system, thus improving the diffusion kinetics of Li^+^ and subsequently boosting the electrochemical performance of the LSBs [54].

To further verify the catalytic effect of the photoelectrode on polysulfide conversion, Li_2_S nucleation experiments were performed with and without the illumination (Fig. 4e). Li_2_S was deposited at a constant potential of 2.10 V with the illumination, whereas in the absence of light, the deposition potential of Li_2_S decreases to 2.04 V, indicating that illumination can reduce the energy barrier for the conversion of polysulfides to Li_2_S. Meanwhile, the Li_2_S nucleation capacity under illumination (493.34 mAh g^−1^) is 71.47 mAh g^−1^ higher than that without the illumination (421.87 mAh g^−1^), indicating a higher conversion rate of polysulfides with illumination. Additionally, the nucleation time of Li_2_S is advanced from 3600 to 3010 s, which further confirms the positive effect of photocatalysis on improving the electrochemical kinetics. The deposition pattern of Li_2_S was revealed by a dimensionless current–time transient (Fig. 4f). During the initial nucleation stage, the precipitation of Li_2_S follows 3D instantaneous (3DI) nucleation mechanism. The nucleation pattern changes with the increase of the amount of deposited Li_2_S. In the absence of light, the nucleation of Li_2_S is corresponding to the 2D progressive (2DP) while the Li_2_S deposition is 2D instantaneous (2DI) model with the illumination [55]. These results can be attributed to the fact that photo-generated carriers increase the surface charge concentration and conductivity of the electrodes, and photo-generated electrons facilitate the transformation of polysulfides to Li_2_S, enabling more Li_2_S to be deposited along the nanorods.

Based on above studies, it is evident that the dynamic processes of PPy@N-TiO_2_/CC assembled PALSBs are significantly accelerated under light illumination. Furthermore, the electrochemical performance of PPy@N-TiO_2_/CC batteries was tested at different current densities. Under illumination, the battery exhibits a reduced polarization voltage and delivers a specific discharge capacity of 1653 mAh g^−1^ at 0.2 C (Fig. S23), which is equivalent to 98.7% of the theoretical capacity (1675 mAh g^−1^), significantly superior to that under dark conditions (1344 mAh g^−1^). The extended second discharge voltage plateau indicates that the photocatalytic effect effectively promotes the conversion of polysulfides to Li_2_S and accelerates the liquid–solid conversion process during the sulfur reduction reaction. In addition, the electrode morphology was observed after the initial discharge (Fig. S24a, b). The discharge products in the absence of light are deposited on the electrode surface in the form of bulk, which hinders the active sites that catalyze the further reaction of polysulfides. In contrast, the reaction products are deposited on the nanorods as thin film sheets, which facilitates the deposition of more Li_2_S along the nanorods. The rate performance of the PALSB was evaluated from 0.5 to 4 C (Fig. 4g). PALSB provides a discharge specific capacity of 1506 mAh g^−1^ at 0.5 C and maintains a discharge capacity of 599 mAh g^−1^ at 4 C with the illumination. When the current drops to 0.5 C, a high capacity of 1403 mAh g^−1^ can still be obtained, verifying the excellent reversibility of the PALSB. In contrast, there is only a discharge specific capacity of 241 mAh g^−1^ at 4 C without the illumination. To explore the influence of photothermal effect on battery capacity enhancement, the discharge capacities at different currents under dark and light conditions were conducted. Under photothermal conditions, the discharge capacities of PPy@N-TiO_2_ assembled LSBs are 1328, 1121, 884, 323, and 244 mAh g^−1^ at 0.5, 1, 2, 3, and 4 C, respectively, which is 20, 15, 34, 5, and 2 mAh g^−1^ higher than that of dark conditions. These results demonstrate that the photothermal effect generated by 60 mW cm^−2^ illumination is not significant for the capacity improvement (Fig. S25). Therefore, the enhancement in electrochemical performance under light illumination can be attributed to the synergistic effects of photocatalytic and photoconductive [27]. Photo-generated carriers not only increase the carrier density of the battery system and enhance the electrochemical kinetics, but also effectively inhibit the shuttle effect and accelerate the sulfur redox reaction, while improving the utilization of active substances.

Figure 4h shows the charge/discharge potential profiles of the PPy@N-TiO_2_/CC assembled PALSB by altering the illumination periodically. It can be observed that the discharge median voltage increased from 2.057 V under dark conditions to 2.121 V under light conditions, while the charge median voltage decreased from 2.335 to 2.312 V under dark conditions (Fig. S26a, b). At the final cycle, an increase of 39 mV in the median discharge voltage and a decrease of 24 mV in the median charging voltage were also observed (Fig. S26c, d). During 90 h of GCD test, the average discharge median potential increased by 40 mV and the average charge median potential decreased by 16 mV under light irradiation compared to dark condition, confirming the photo-generated carrier accelerated sulfur redox kinetics performance in the presence of light. It is worth noting that the enhancement effect of photo-assisted effect in the discharge process is more obvious, which has a strong correlation to the band structure of the photoelectrode. Specifically, the potential of the sulfur redox reaction is closer to the CB than the VB of the photoelectrode. Figure 4i illustrates the cycling performance of PALSBs. The reversible capacity achieves 1048 mAh g^−1^ during 69 cycles, which is 308 mAh g^−1^ higher than that without illumination (740 mAh g^−1^). Furthermore, the morphology of the electrodes after 69 cycles under different environment was observed (insets of Fig. 4i). Numerous irregular coverings are deposited on the surface of the PPy@N-TiO_2_/CC electrode without the illumination while most of the active sites are exposed under the light illumination, showing better electrochemical reversibility and stability. Even at a high sulfur loading of 3 mg cm^−2^, the discharge specific capacity of 864 mAh g^−1^ can be achieved in the first cycle under illumination, while the discharge specific capacity can only reach 587 mAh g^−1^ under dark condition (Fig. S27). The maximum areal capacity of PALSB reaches 3.3 mAh cm^−2^, and maintains excellent capacity retention over 24 cycles (Fig. 4j), indicating the practical application potential of PPy@N-TiO_2_/CC assembled PALSB. The capacity fluctuations observed under light conditions during cycling are closely related to the light power density. During prolonged operation, the fluctuation of optical power density of xenon lamp light source affects the photocatalytic performance of photoelectrode, thus showing the variations of capacity. To investigate the mechanism of capacity enhancement during photo-assisted process, the cycling performance of the battery assembled with PPy@N-TiO_2_/CC electrode (without Li_2_S_6_) was evaluated at a current density of 0.1 C under light conditions (Fig. S28). The test results demonstrate that PPy@N-TiO_2_/CC battery delivered a capacity below 20 mAh g^−1^ during cycling, confirming that the contribution from non-faradaic capacitance is almost negligible.

**Fig. 5:**
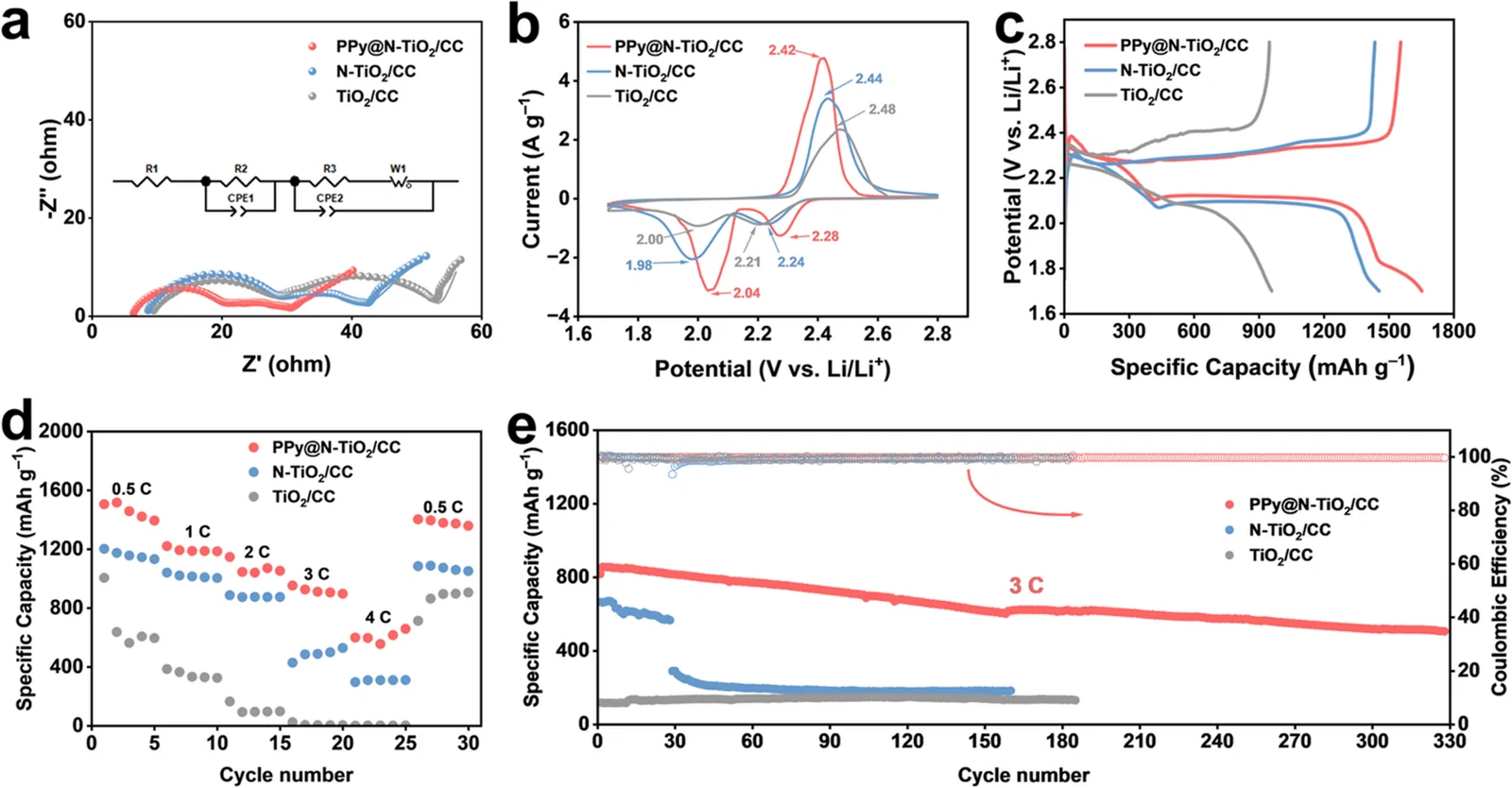
Electrochemical performance of PALSBs with various photoelectrodes under illumination: **a** EIS curves; **b** CV curves at 0.1 mV s^−1^; **c** GCD curves at 0.2 C; **d** rate performance;** e** cycling performance with the illumination at 3 C

To further investigate the active role of the p–n junction formed by PPy@N-TiO_2_/CC in the catalytic process, the electrochemical performance of PALSB was tested by assembling with TiO_2_/CC, N-TiO_2_/CC, and PPy@N-TiO_2/_CC photoelectrodes, respectively. As shown in Fig. 5a, the PPy@N-TiO_2/_CC battery exhibits smaller interface contact resistance and charge transfer impedance, which can be attributed to the introduced synergistic effect between N-doping and PPy. The CV curves also demonstrate the superiority of the p–n junction in the catalytic process, showing the smallest polarization voltage and the corresponding maximum peak current (Fig. 5b). In addition, the redox peaks of CV curves were fitted by Tafel (Fig. S29). It can be concluded that the PPy@N-TiO_2_/CC battery exhibits the best catalytic ability under light illumination. These results indicate that the constructed p–n junction can effectively enhance the separation efficiency of photo-generated carriers based on the IEF, thereby greatly improving the carrier concentration of the system and the electrochemical kinetic performance of the battery through photoconductivity and photocatalysis. As expected, the PALSB assembled with PPy@N-TiO_2_/CC photoelectrode shows the highest discharge capacity (1653 mAh g^−1^) and the best rate performance compared to N-TiO_2_/CC (1455 mAh g^−1^) and TiO_2_/CC (959 mAh g^−1^) PALSBs (Fig. 5c, d). Moreover, the photo-assisted PPy@N-TiO_2_/CC battery can achieve a maximum discharge specific capacity of 856 mAh g^−1^ at 3 C while only 342 mAh g^−1^ is obtained under dark conditions. The superior photocatalytic activity also enables a low capacity decay rate of only 0.12% per cycle after 328 cycles (Figs. 5e and S30). In contrast, the photo-assisted N-TiO_2_/CC battery provides a capacity of 664 mAh g^−1^ in the initial cycle, and then decays rapidly, which may be attributed to the decrease in photocatalytic activity. Meanwhile, the photo-assisted TiO_2_/CC battery exhibits no obvious response to illumination.

### Photoelectric Conversion Performance of PPy@N-TiO_2_/CC Photoelectrode

**Fig. 6:**
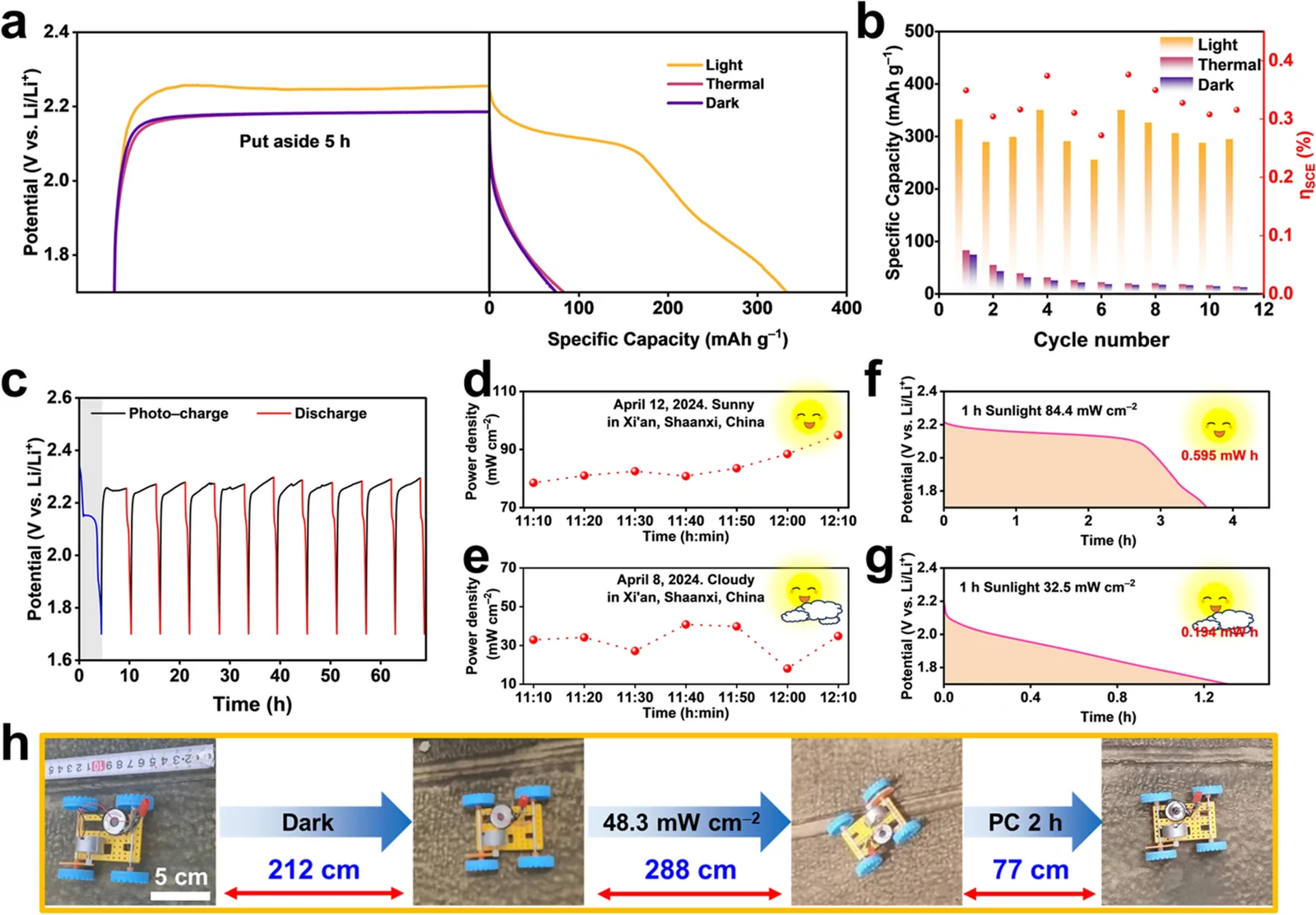
**a** Discharge curves at 0.2 C after put aside 5 h at different conditions. **b** Cycling performance and solar conversion efficiency of PPy@N-TiO_2_/CC PALSB at 0.2 C after 5 h of photo-charging. **c** Discharge curves at 0.2 C after 5 h of photo-charging. **d, e** Photo power density in different weather conditions. The discharge curves after 1 h of photo-charging on **f** sunny and **g** cloudy days. **h** Evaluation of the practicability of PPy@N-TiO_2_/CC PALSB by equipped in a toy car

To further explore the practical application potential of the PPy@N-TiO_2_/CC PALSB, photoelectric conversion performance of the energy storage system was evaluated by directly photo-charging. The completely discharged LSB was exposed to illumination without external voltage. As a result, the battery reached a voltage of 2.25 V and achieved a high discharge specific capacity of 333 mAh g^−1^ at 0.2 C after 5 h of photo-charging, exceeding the level of conventional lithium-ion batteries. On the contrary, the voltage of the fully discharged LSB can only rise to approximately 2.18 V and provide a discharge capacity of less than 85 mAh g^−1^ when put aside for 5 h under dark/thermal conditions (Fig. 6a), which is attributed to the inherent polarization effect inside the battery. To quantify the charge stored during the photo-charging process of the PALSB, the ratio of the discharge energy to the input solar energy in the active area is defined as the overall solar energy conversion efficiency (*η*_SCE_). The *η*_SCE_ of the PALSB over 11 cycles is about 0.33%, implying the great application potential of PALSB (Fig. 6 b,c).

To further verify the feasibility of the practical application of PPy@N-TiO_2_/CC PALSB, the photoelectric conversion ability was evaluated on sunny and cloudy days, respectively. The recorded photo power density is shown in Fig. 6d, e. Under different conditions, the PALSB after 1 h of sunlight charging delivers discharge energy of 0.595 and 0.194 mWh with *η*_SCE_ of 1.41% and 1.19%, respectively (Fig. 6f, g). The cycling performance of the PALSB with a sulfur loading of 3 mg cm^−2^ was evaluated by simulating a sunny environment. A high areal capacity of 2.7 mAh cm^−2^ at 0.5 C was obtained under 84.4 mW cm^−2^ of sunlight, and it could last for more than 25 cycles (Fig. S31). These results show that the PALSB can play its unique advantages in different weather conditions. Furthermore, an electronic thermometer equipped with a PPy@N-TiO_2_/CC assembled 2032-coin cell was used to explore its usability (Fig. S32). When fully charged, the electronic thermometer operated for over 60 h. The PALSB in the fully discharged state was placed under dark conditions, and the electronic thermometer could not work. Subsequently, the PALSB in the fully discharged state was exposed to light for direct photo-charging. Interestingly, the electronic thermometer can work again. Then, the application scenario of the PPy@N-TiO_2_/CC PALSB was extended. As shown in Fig. 6h, it can be observed that just one coin PALSB can drive the toy car forward about 212 cm (Video S1). Due to the photo-assisted discharge effect, the driving distance of the toy car is extended to approximately 288 cm (Video S2). Encouragingly, when the power of PALSB ran out, the PALSB could still drive the toy car 77 cm after 2 h of photo-charging (Video S3). These results indicate that the PALSB realizes the integration of solar energy conversion and storage, making it highly suitable for application in high-altitude and aviation environments owing to its unique advantages.

## Conclusion

In summary, a free-standing PPy@N-TiO_2_/CC multifunctional photoelectrode with a direct Z-scheme heterojunction is designed for advanced photo-assisted lithium–sulfur batteries (PALSBs). The PPy@N-TiO_2_/CC photocathode serves dual functions: As an electrocatalyst, it promotes the conversion of intermediate polysulfides, effectively suppressing side reactions during charge/discharge cycles. Simultaneously, as a photocatalyst, it accelerates sulfur reduction and evolution through synergistic photocatalytic, photoconductive, and photo-charging effects under light illumination. The p–n heterojunction formed between N-TiO_2_ and PPy generates a robust internal electric field (IEF), which significantly enhances the separation of photo-generated carriers and improves solar energy utilization efficiency. Owing to the synergistic coupling of photocatalysis and electrocatalysis, the PPy@N-TiO_2_/CC-based PALSB demonstrates remarkable electrochemical performance, including a high specific capacity of 1653 mAh g⁻^1^, exceptional rate capability (599 mAh g⁻^1^ at 4 C), and long-term cycling stability (61.7% capacity retention after 328 cycles). Notably, the PALSB can be directly photo-charged without external bias, achieving a high capacity of 333 mAh g⁻^1^ after 5 h of photo-charging and an overall solar energy conversion efficiency of 0.33%, which surpasses conventional lithium-ion batteries. This work not only advances the development of high-performance PALSBs but also establishes a new paradigm for integrating solar energy harvesting and storage, offering a transformative approach for next-generation sustainable energy systems.

## Supplementary Information

Below is the link to the electronic supplementary material.

Supplementary file1 (MP4 1945 KB)

Supplementary file2 (MP4 3073 KB)

Supplementary file3 (MP4 1154 KB)

Supplementary file4 (DOCX 6874 KB)
