# Supplementary material for: Ultrafast Sulfur Redox Dynamics Enabled by a PPy@N-TiO2 Z-Scheme Heterojunction Photoelectrode for Photo-Assisted Lithium–Sulfur Batteries
Source: Nanomicro Lett. 2026 Jan 1;18:92. doi: 10.1007/s40820-025-01946-3 (PMC12756215; doi:10.1007/s40820-025-01946-3)
Supplement: Supplementary file 4 — Supplementary file4 (DOCX 6874 KB) [file 40820_2025_1946_MOESM4_ESM.docx]

Supporting Information for

**Ultrafast Sulfur Redox Dynamics Enabled by a PPy@N-TiO****_2_ Z-Scheme Heterojunction Photoelectrode for Photo-assisted Lithium-Sulfur Batteries**

Fei Zhao^1, 2^, Yibo He^1, 2,^ *, Xuhong Li^1^, Ke Yang^1, 2^, Shuo Chen^1, 2^, Yuanzhi Jiang^3^, Xue-Sen Wang^4^, Chunyuan Song^1, 2^, and Xuqing Liu^1, 2^

^1^ State Key Laboratory of Solidification Processing, Center of Advanced Lubrication and Seal Materials, School of Materials Science and Engineering, Northwestern Polytechnical University, Xi’an, Shaanxi 710072, P. R. China

^2^ Research & Development Institute of Northwestern Polytechnical University in Shenzhen, Shenzhen 518063, P. R. China

^3^ Key Laboratory of Advanced Energy Materials Chemistry (Ministry of Education), Nankai University, Tianjin 300071, P. R. China

^4^ Department of Physics, National University of Singapore, Singapore 117543, Singapore

*Corresponding author. E-mail: [heyibo@nwpu.edu.cn](mailto:heyibo@nwpu.edu.cn) (Yibo He)

**Supplementary Figures and Table**


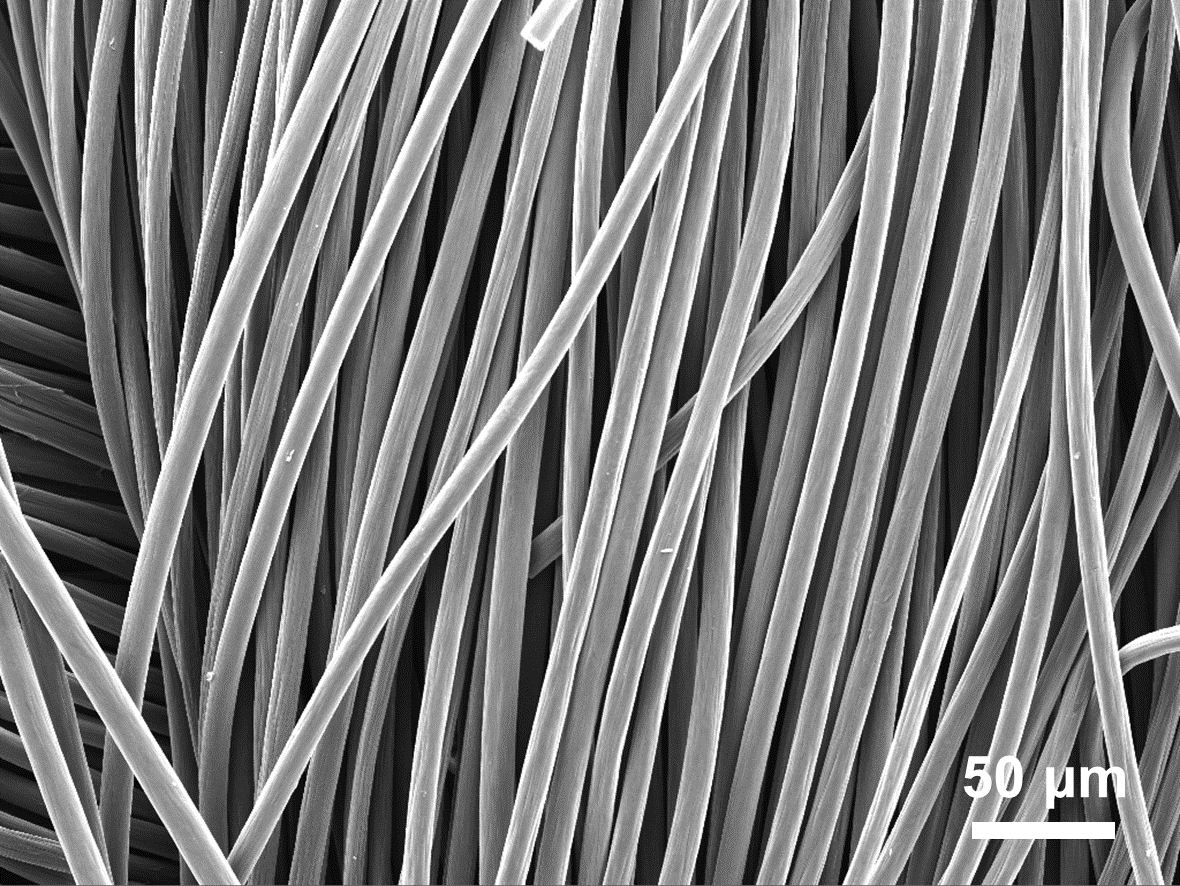


Fig. S1 Low-magnification SEM image of CC


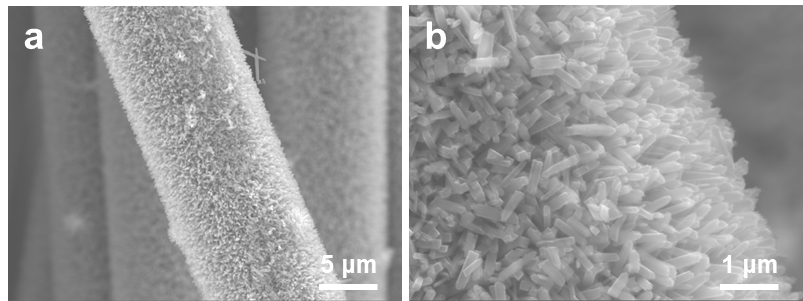


Fig. S2 SEM image of TiO_2_/CC


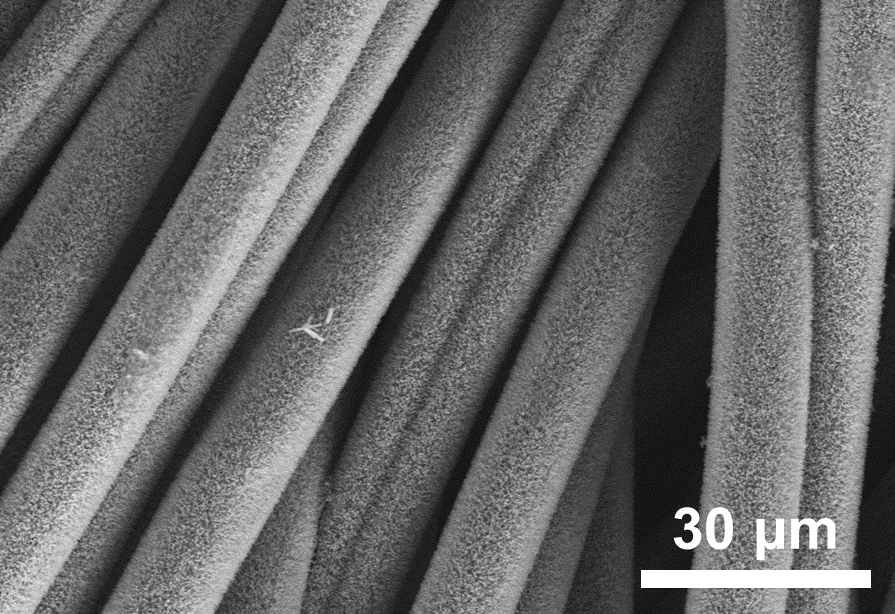


Fig. S3 Low-magnification SEM image of PPy@N-TiO_2_/CC


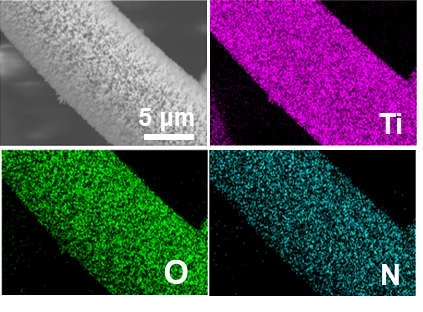


Fig. S4 Element mapping of PPy@N-TiO_2_/CC


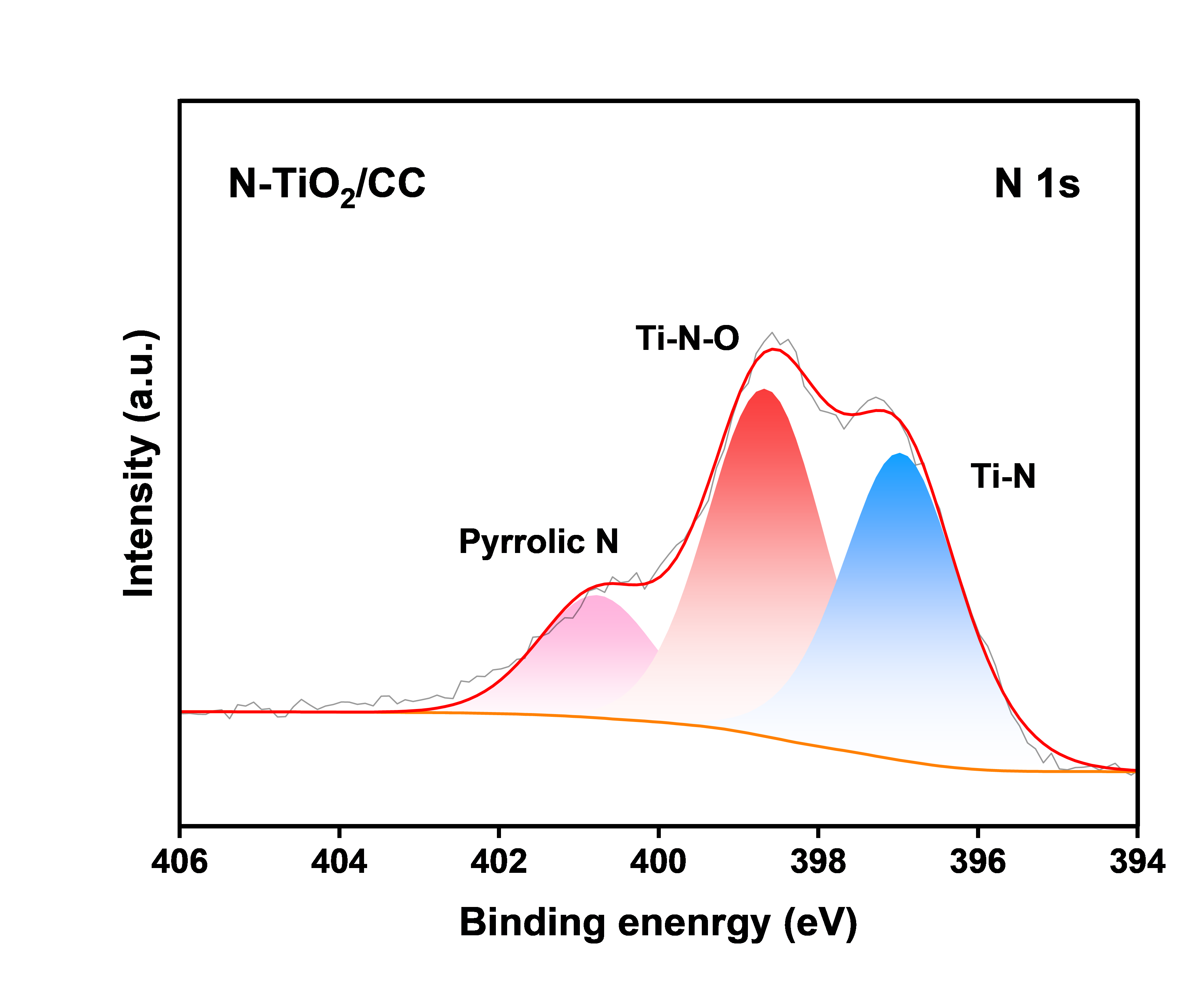


Fig. S5 N 1s XPS spectrum of N-TiO_2_/CC


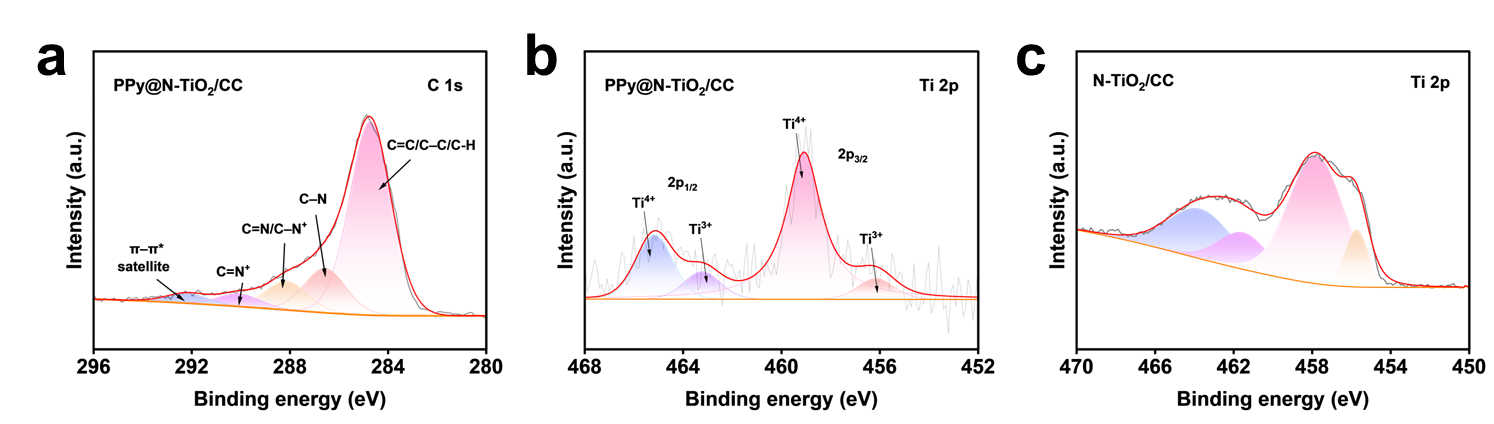


Fig. S6 a C 1s, b Ti 2p XPS spectra of PPy@N-TiO_2_/CC


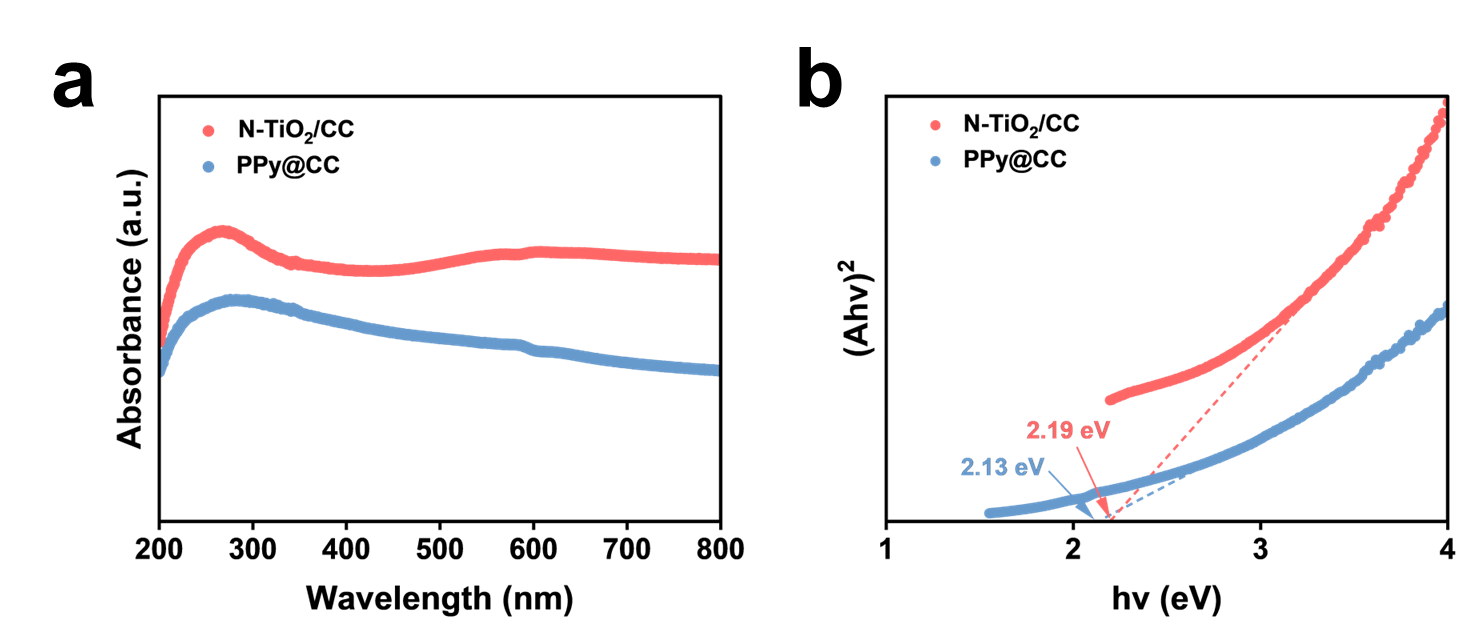


**Fig. S7** **a** UV-vis absorption spectra and **b** the band gap of N-TiO_2_/CC and PPy@CC


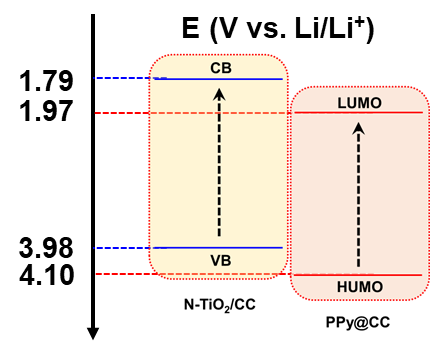


Fig. S8 Band energy diagram of N-TiO_2_/CC and PPy@CC vs. Li/Li^+^


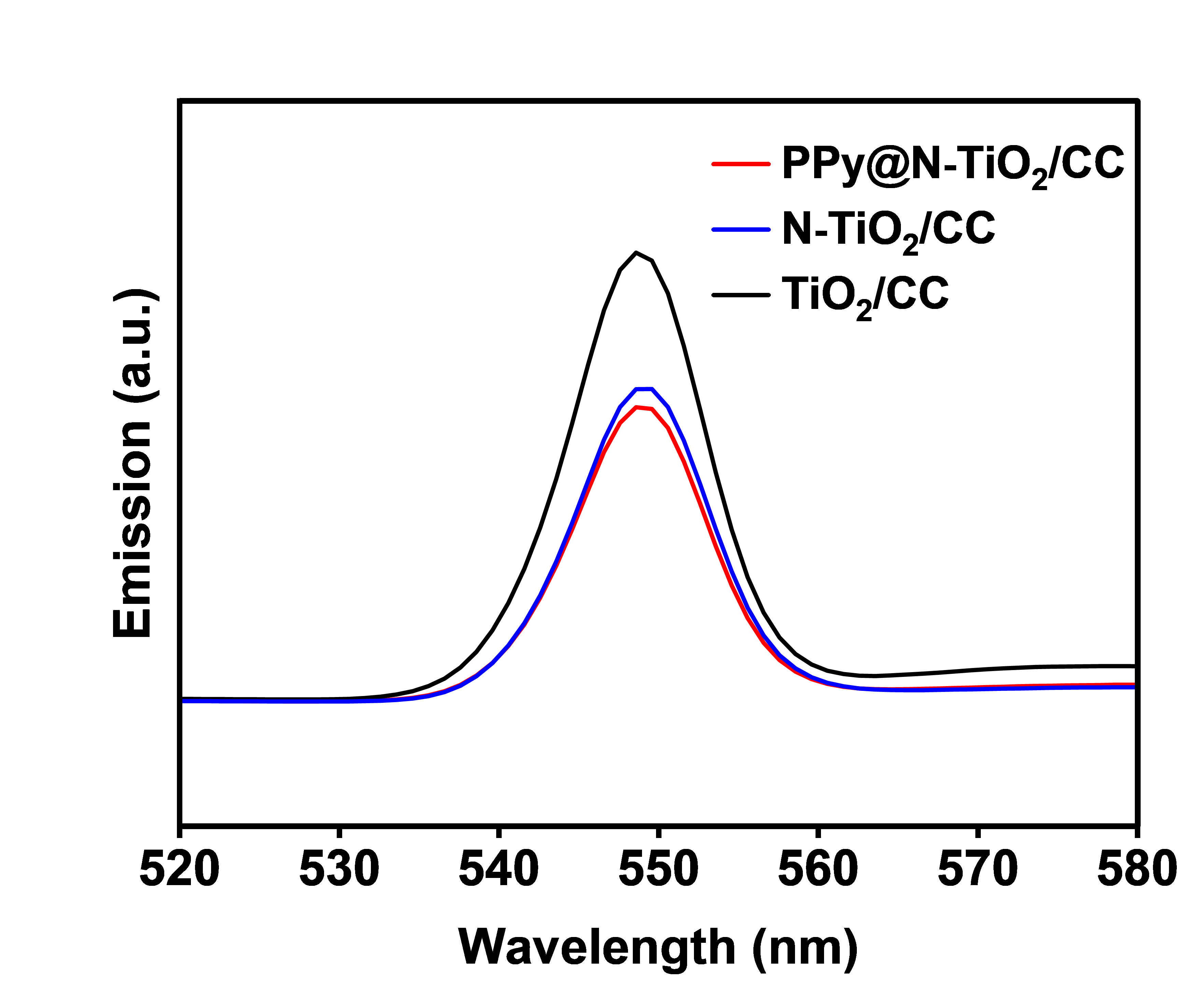


Fig. S9 PL emission spectra of various samples


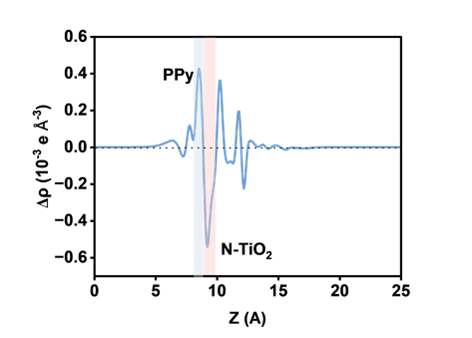


Fig. S10 Average planar electron density difference Δρ(z) for PPy@N-TiO_2_


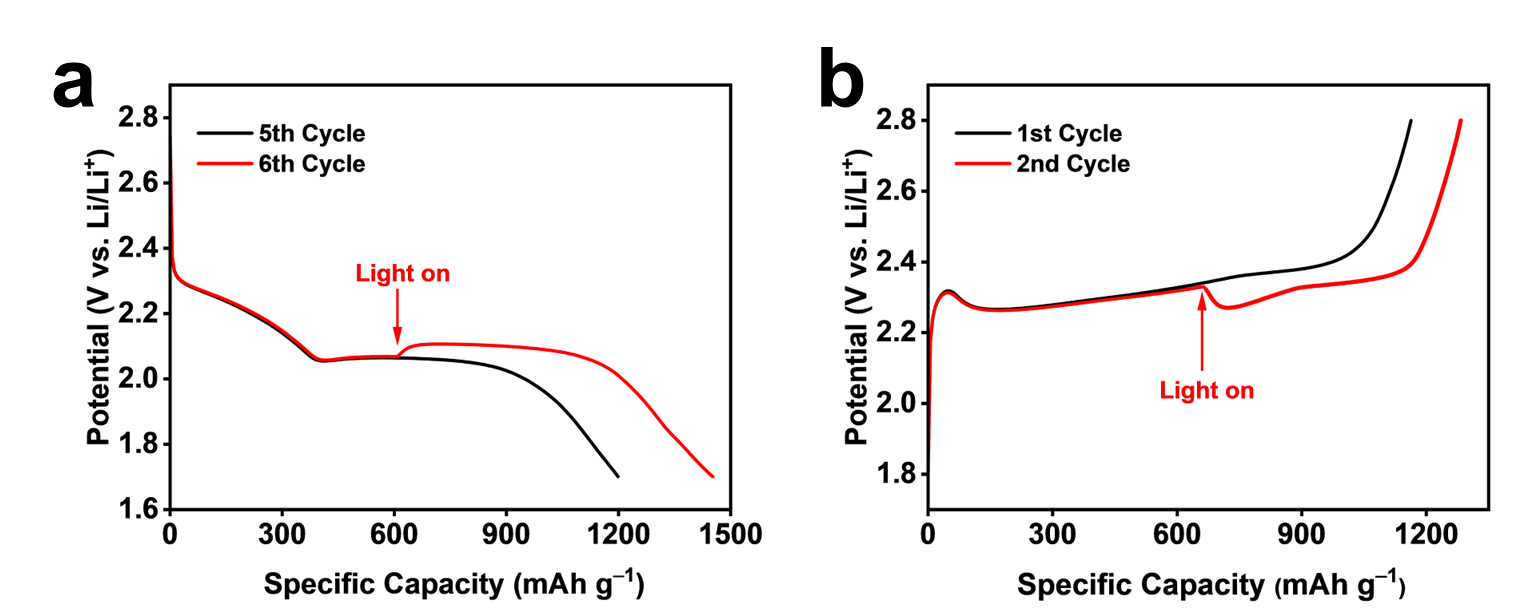


Fig. S11 a Discharge and b charge curves of PPy@N-TiO_2_/CC battery at 0.2 C by altering the light on/off


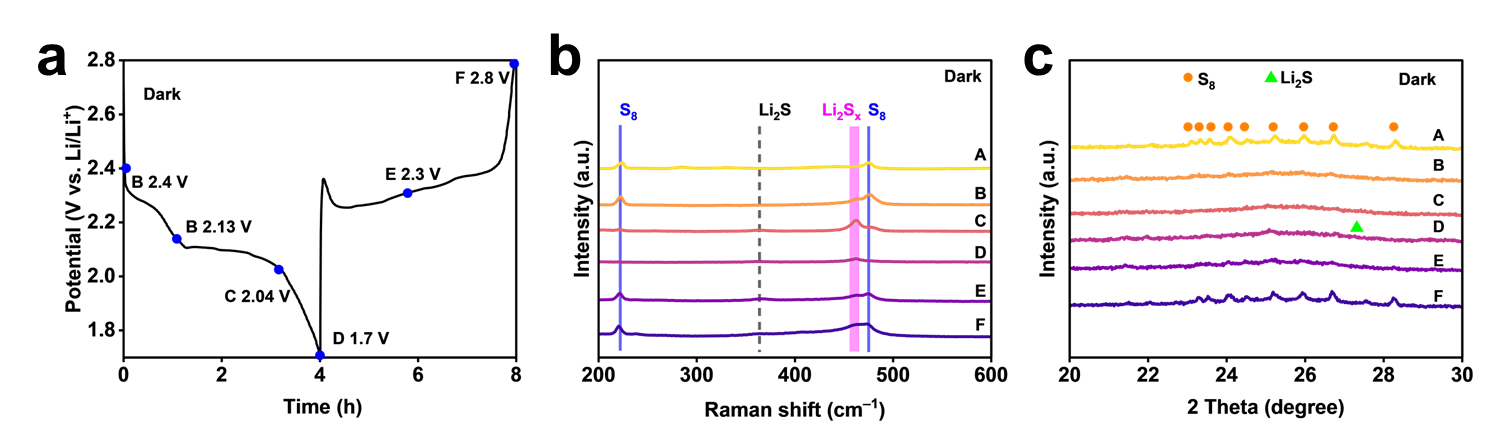


**Fig. S12 a** GCD curves of PPy@N-TiO_2_/CC battery at 0.2 C without the illumination. Corresponding **b** Raman spectra and **c** XRD patterns of PPy@N-TiO_2_/CC photocathode without the illumination


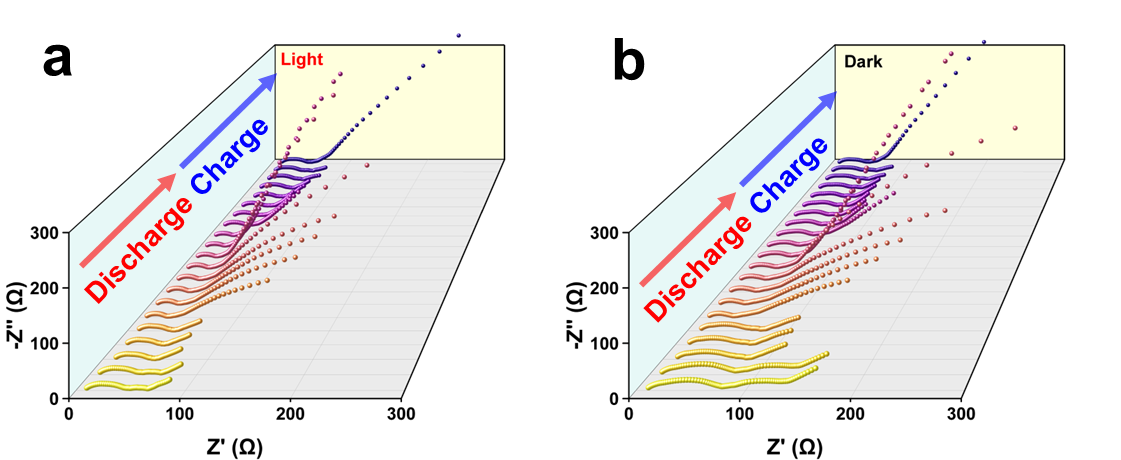


Fig. S13 EIS spectra of the PPy@N-TiO_2_ battery at 0.2 C (a) with and (b) without the illumination

**
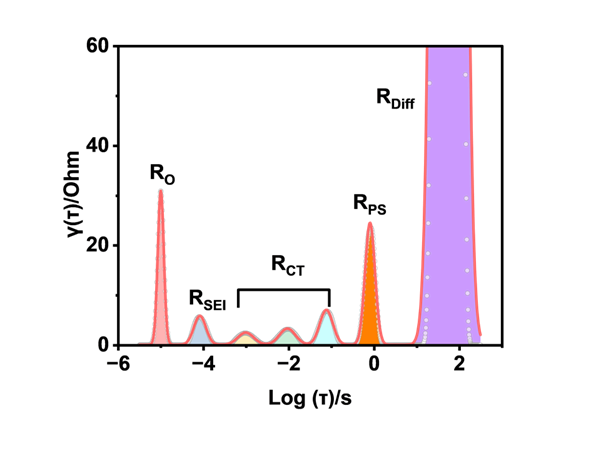
**

**Fig. S14** Schematic diagram of DRT analysis


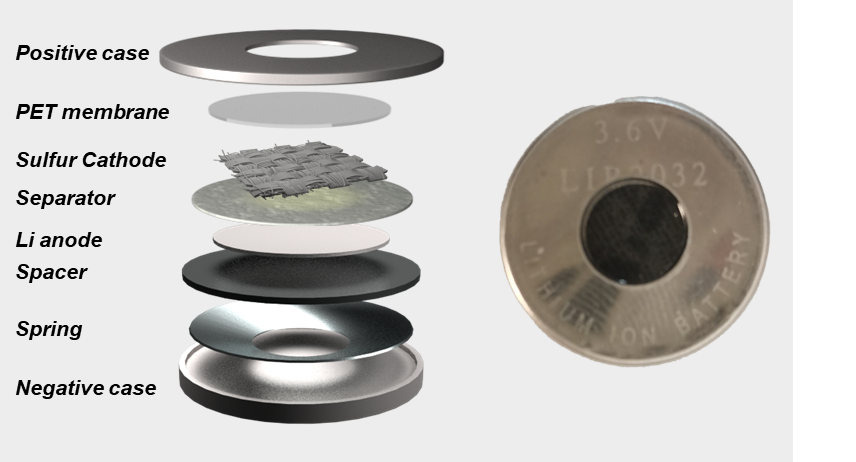


Fig. S15 Schematic diagram and optical photograph of PALSB assembly


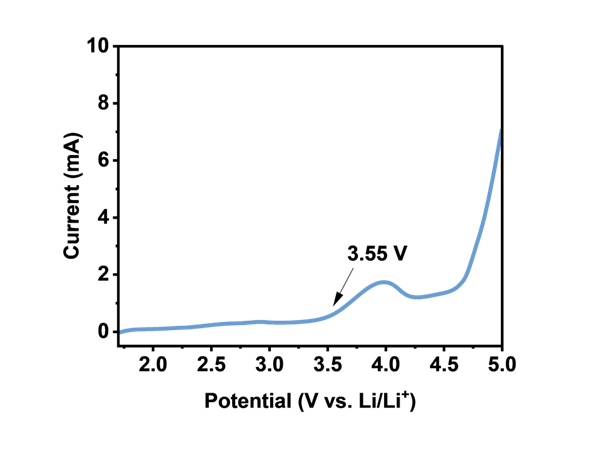


Fig. S16 LSV curves of Li||PPy@N-TiO_2_/CC battery with the illumination


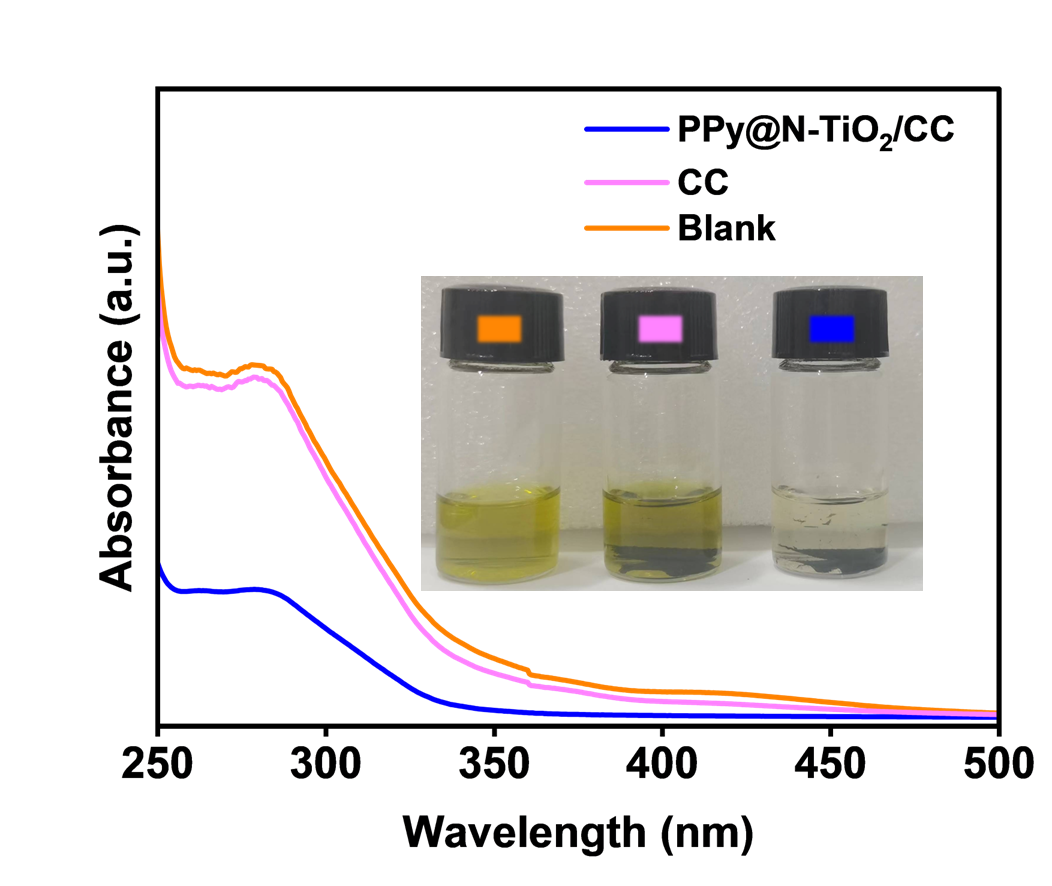


**Fig. S17** UV-vis absorption spectra of Li_2_S_6_ solution with different samples (inset: the digital photo of polysulfides adsorption)


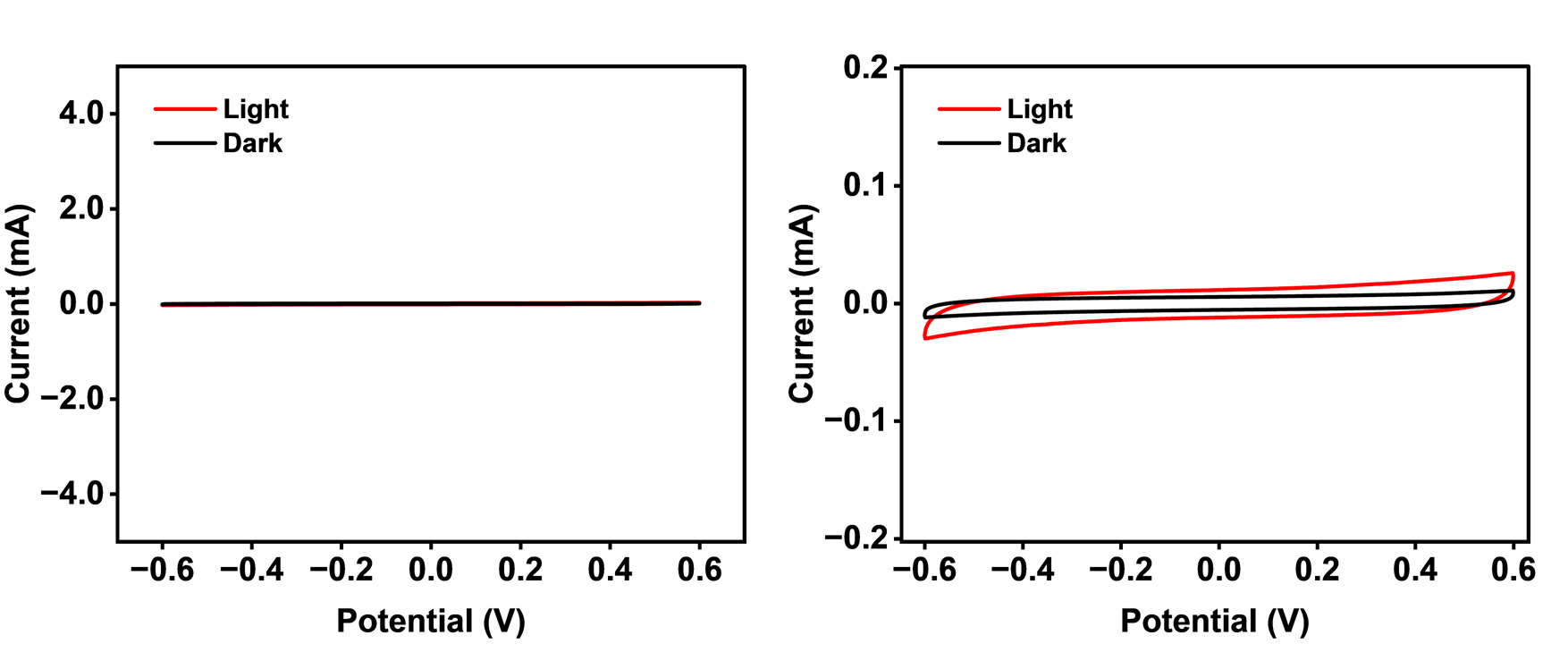


Fig. S18 CV curves of symmetric batteries without Li_2_S_6_ at 0.5 mV s^–1^ with PPy@N-TiO_2_/CC electrode with and without the illumination


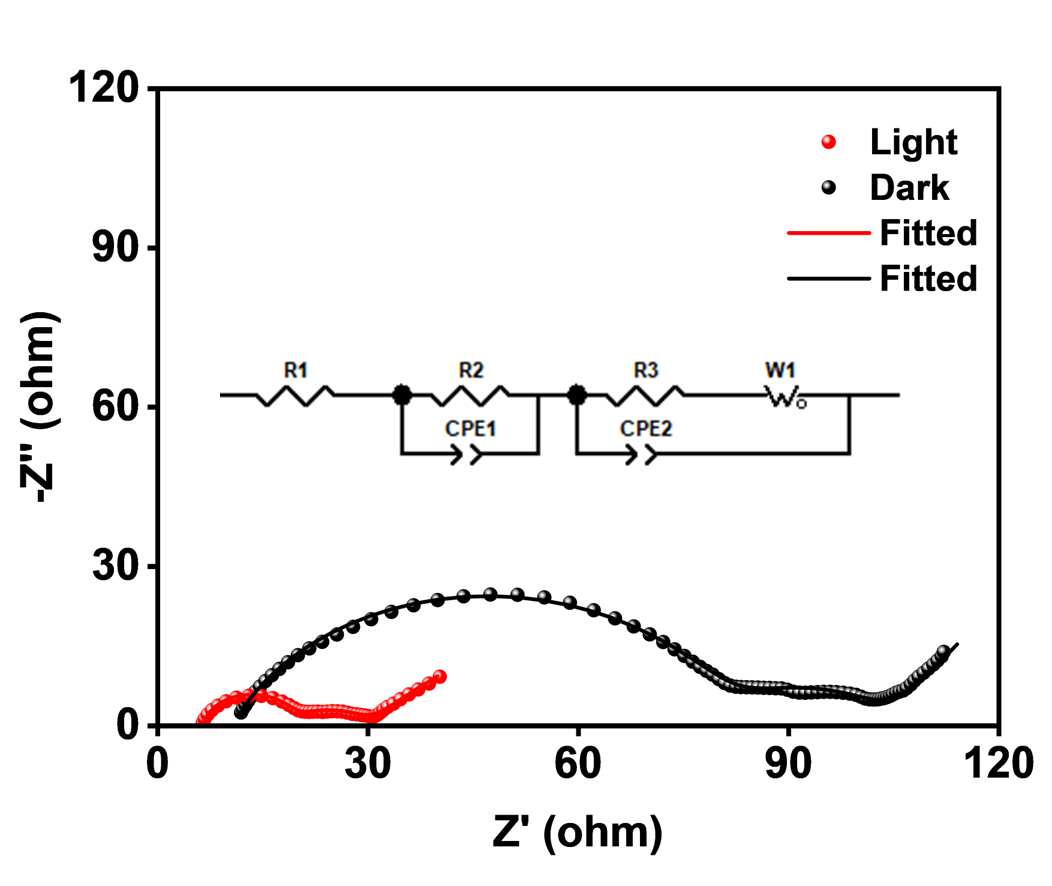


Fig. S19 EIS curves of PPy@N-TiO_2_/CC assembled PALSB with and without the illumination


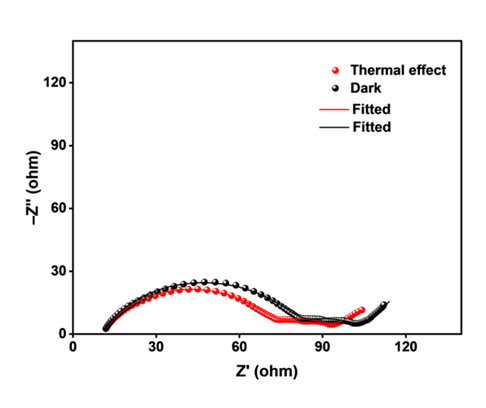


Fig. S20 EIS curves of PPy@N-TiO_2_/CC assembled PALSB under photothermal


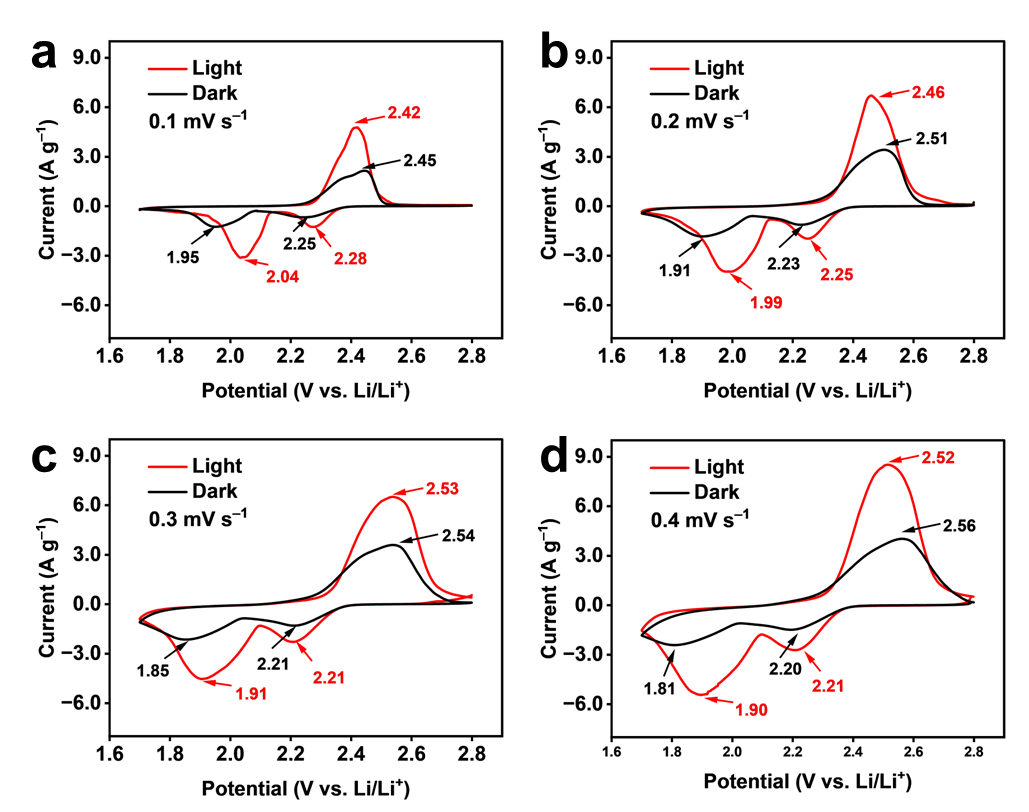


Fig. S21 CV curves of PPy@N-TiO_2_/CC battery with and without the illumination at a 0.1 mV s^–1^, b 0.2 mV s^–1^, c 0.3 mV s^–1^, and d 0.4 mV s^–1^
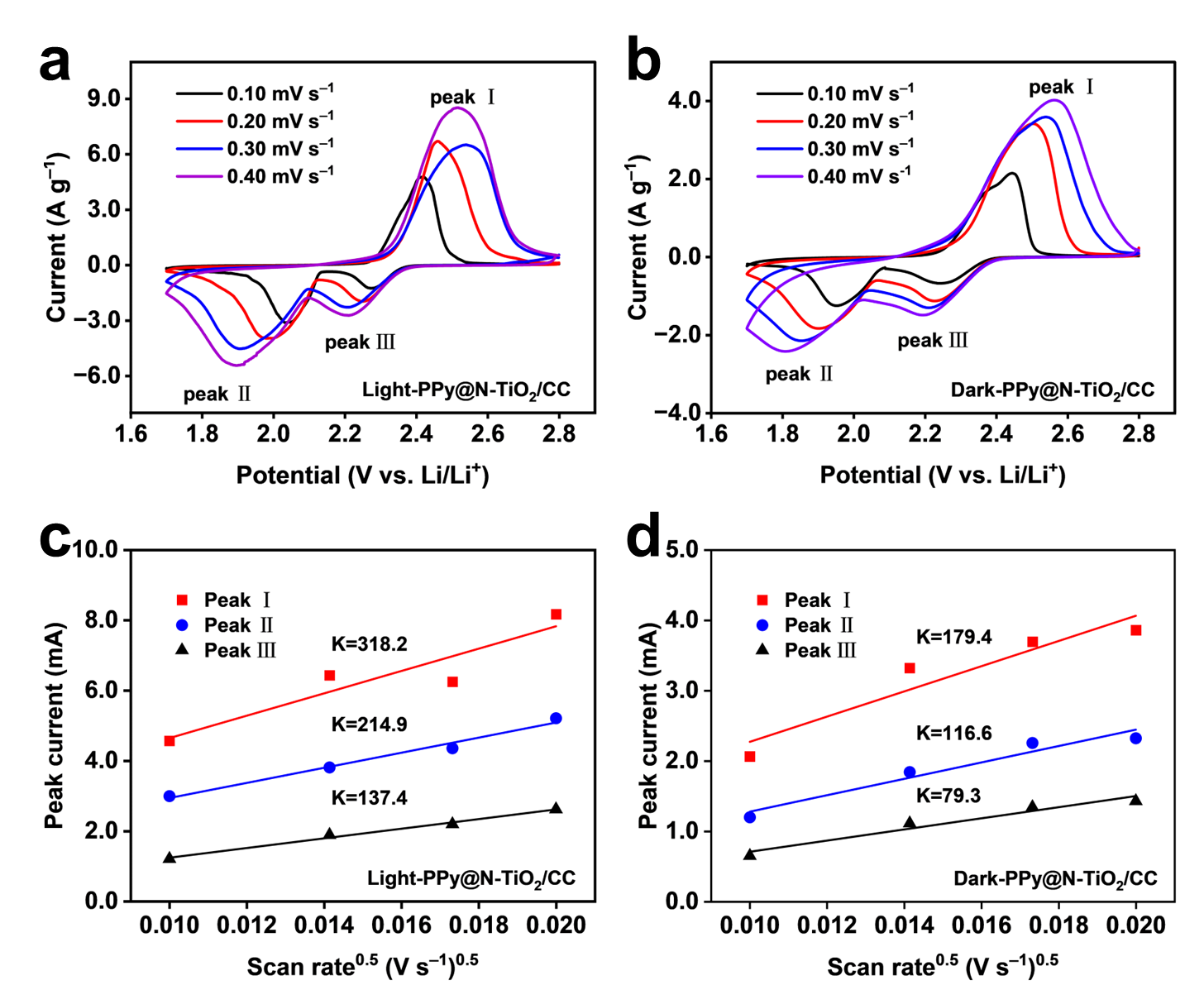


Fig. S22 CV curves of PPy@N-TiO_2_/CC battery a with and b without the illumination at different scan rates. Plots of CV peak current versus square root of scan rate c with and d without the illumination


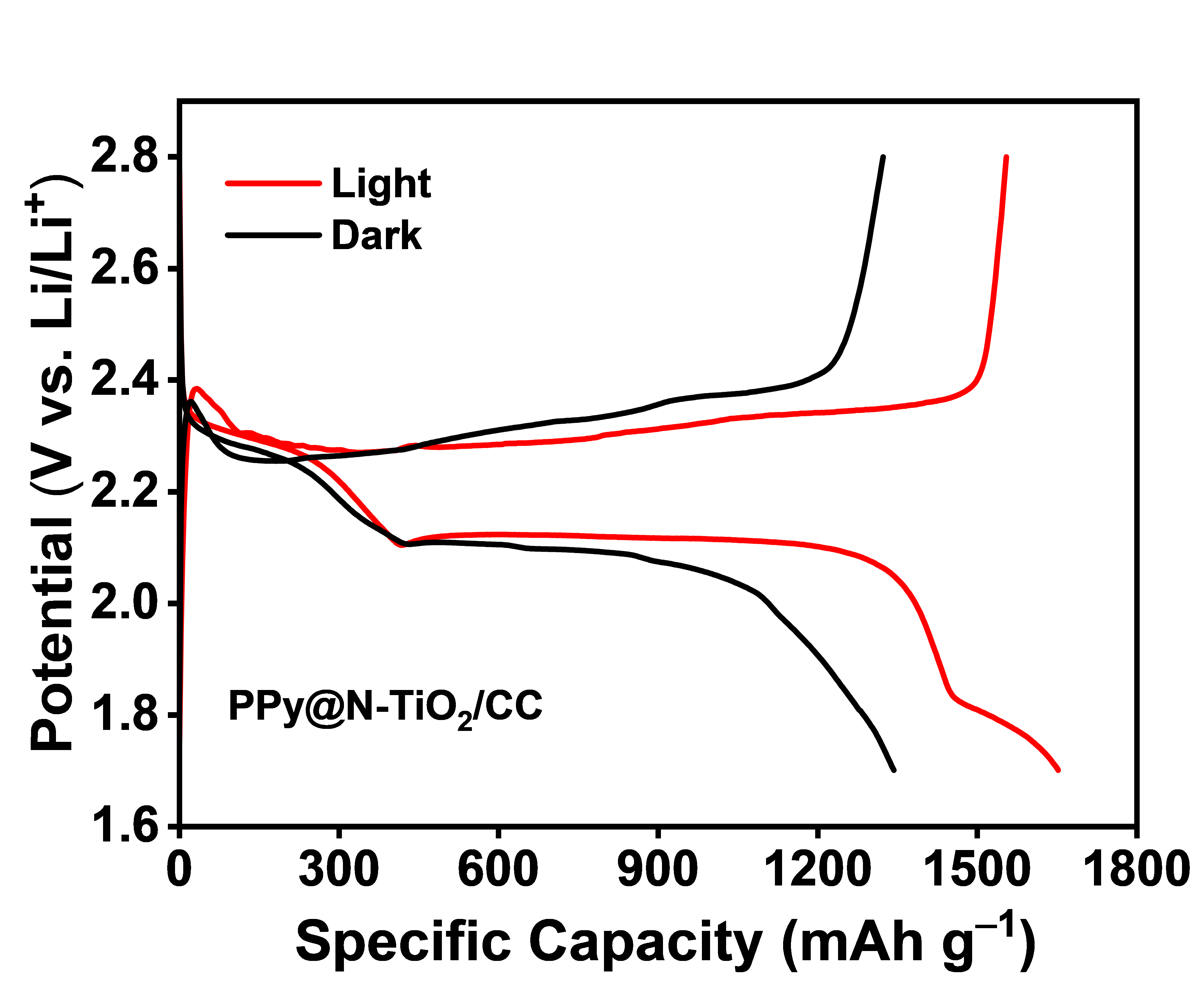


Fig. S23 GCD curves of PPy@N-TiO_2_/CC battery at 0.2 C with and without the illumination


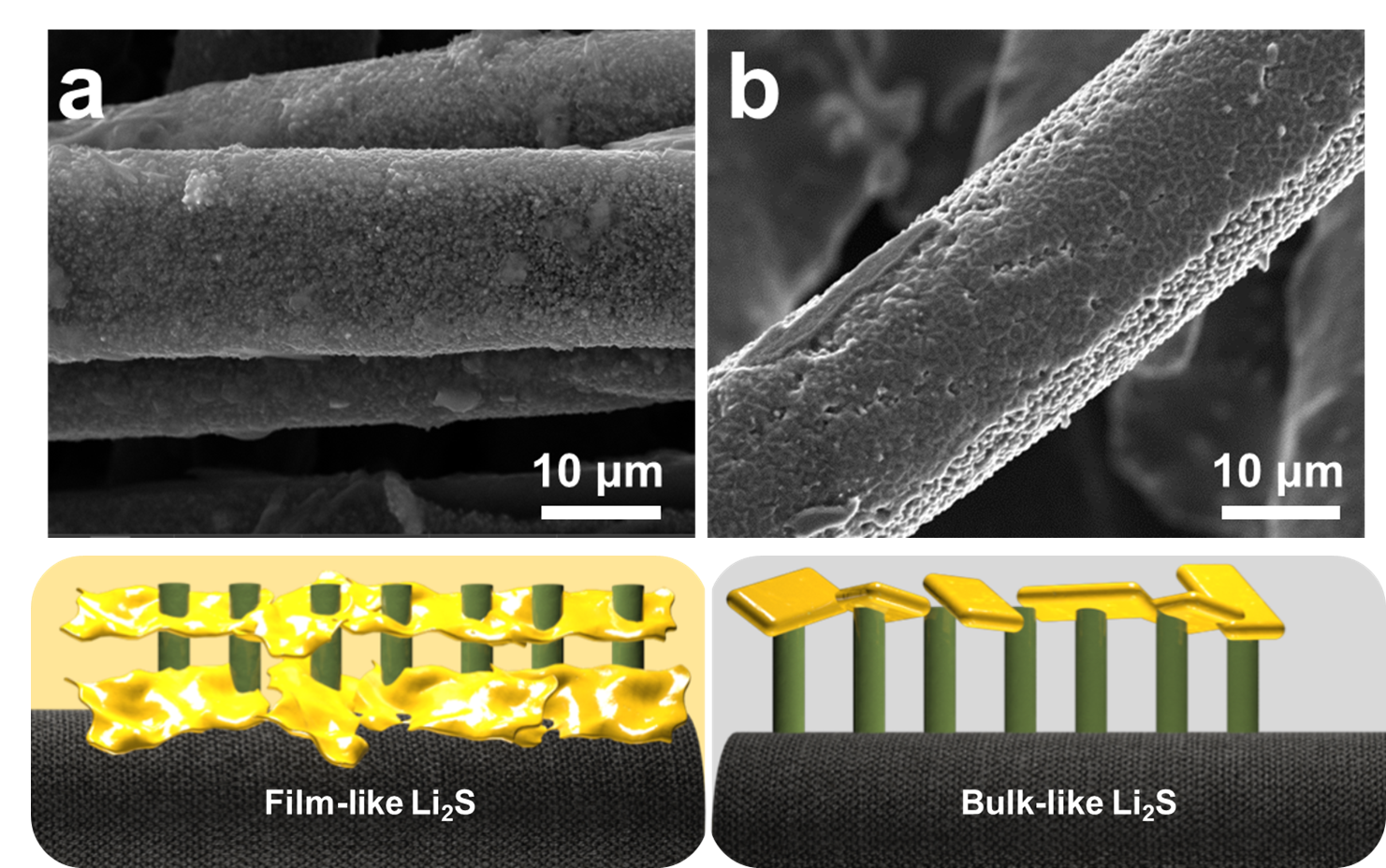


Fig. S24 The SEM images of PPy@N-TiO_2_/CC electrode after being initially discharged a with and b without the illumination (Schematic diagram of discharge product morphology with and without the illumination)


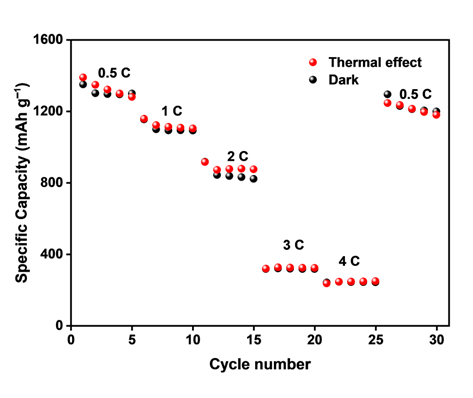


Fig. S25 Rate performance of PPy@N-TiO_2_/CC battery under photothermal


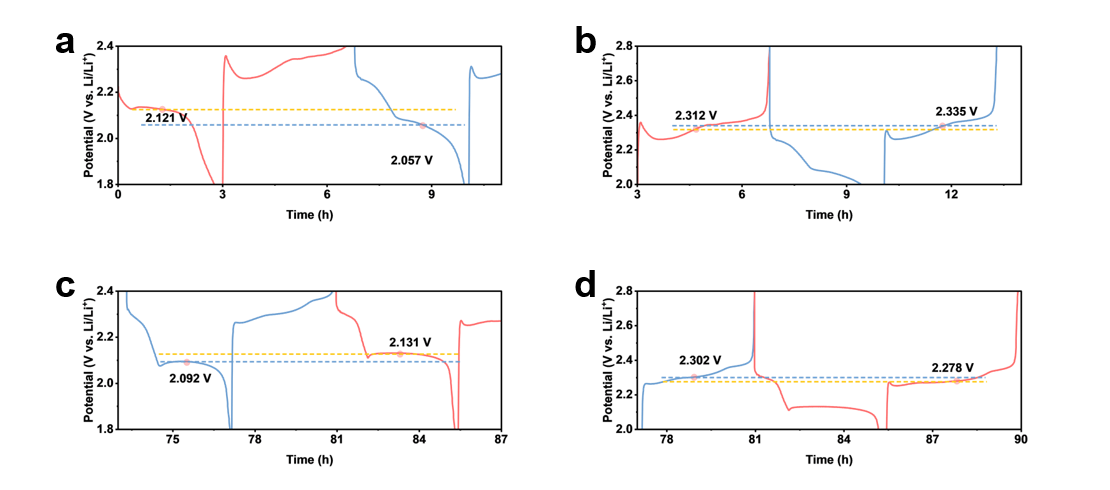


Fig. S26 Partially enlarged GCD curves by altering the illumination periodically: a,b initial cycle, c,d final cycle


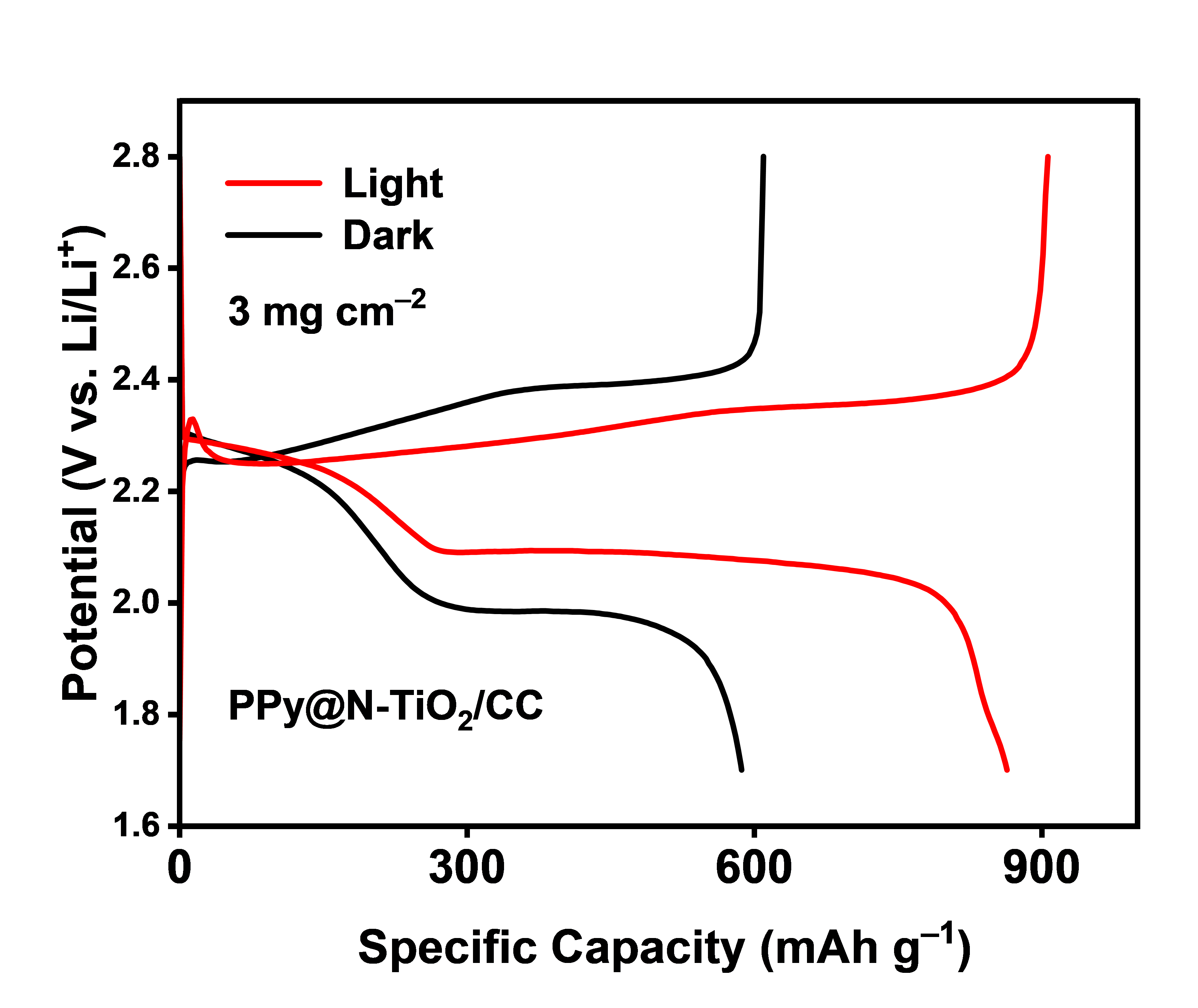


**Fig. S27** GCD curves with sulfur loading of 3 mg cm^–2^ at 0.2 C


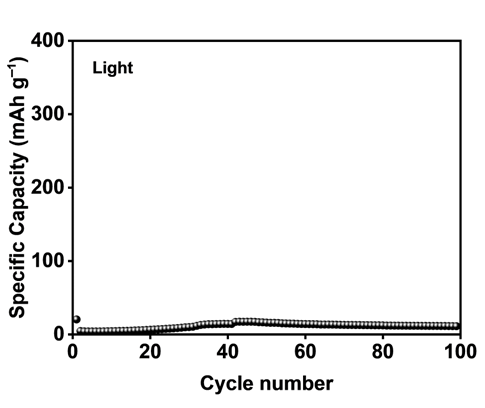


**Fig. S28** Cycling performance of PPy@N-TiO_2_/CC without the Li_2_S_6_ solution


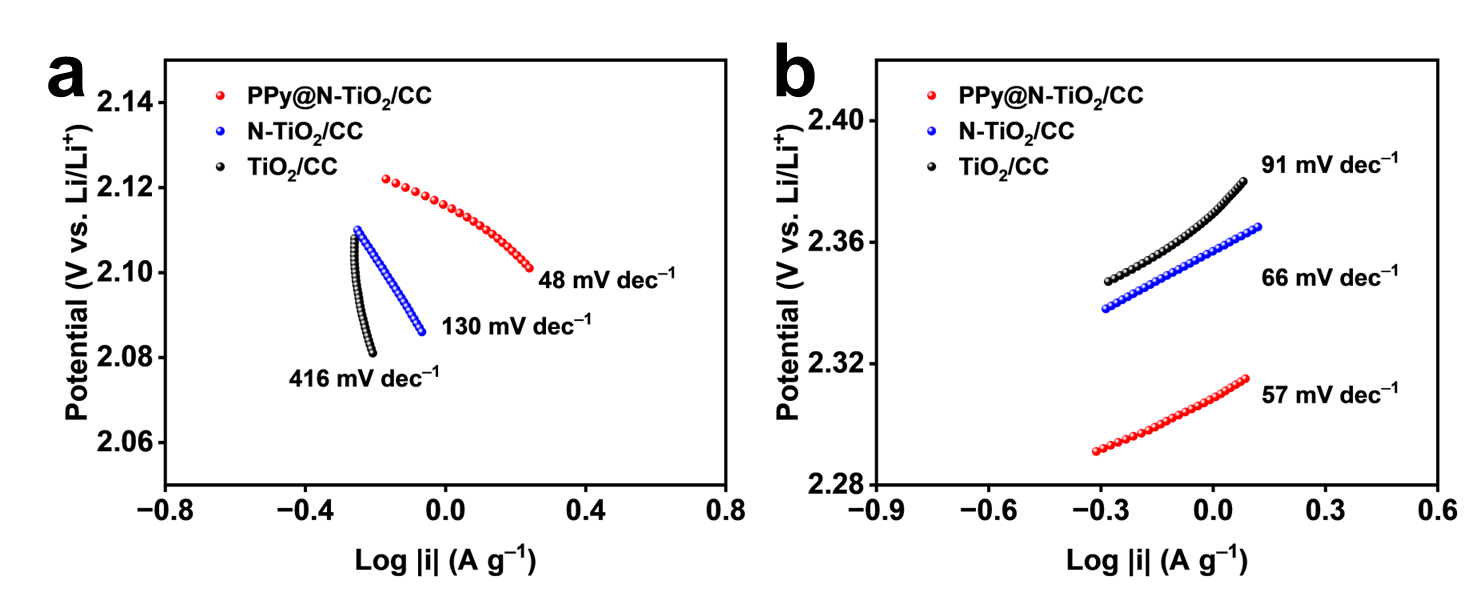


Fig. S29 a, b Tafel curves of PPy@N-TiO_2_/CC, N-TiO_2_/CC and TiO_2_/CC electrodes with the illumination


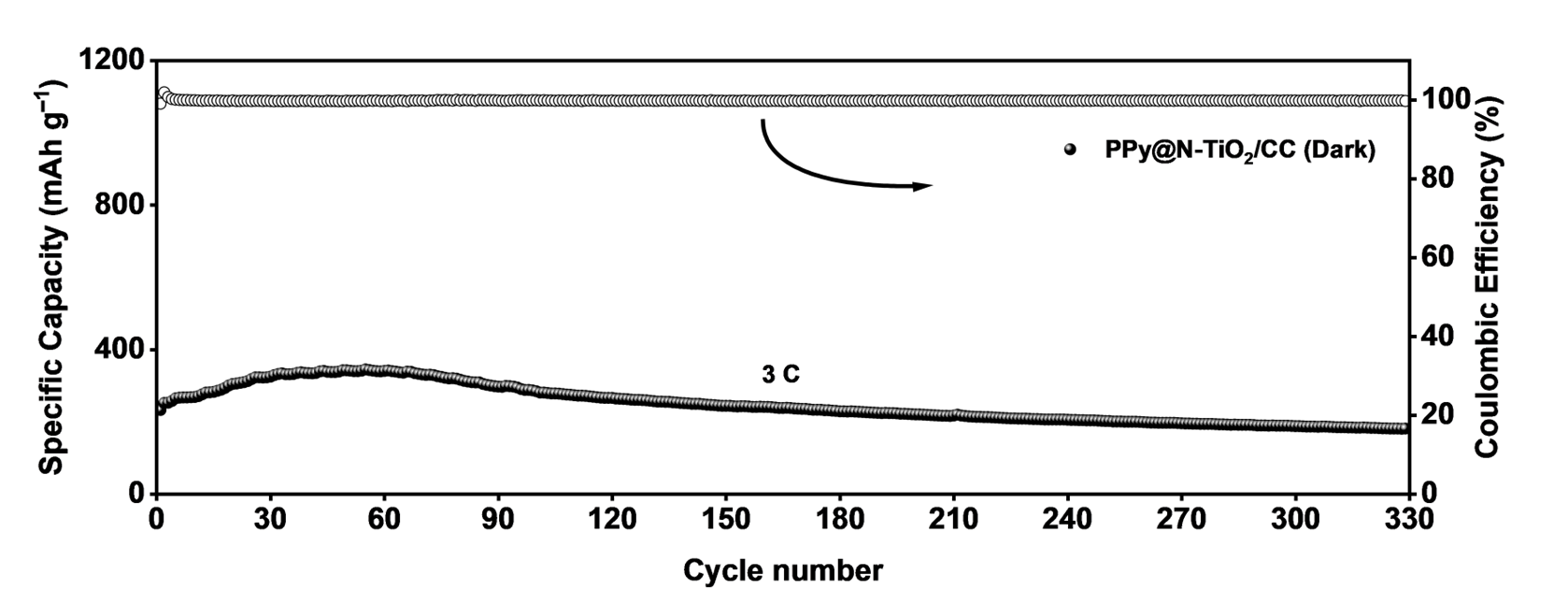


Fig. S30 Cycling performance of PPy@N-TiO_2_/CC battery at 3 C without the illumination


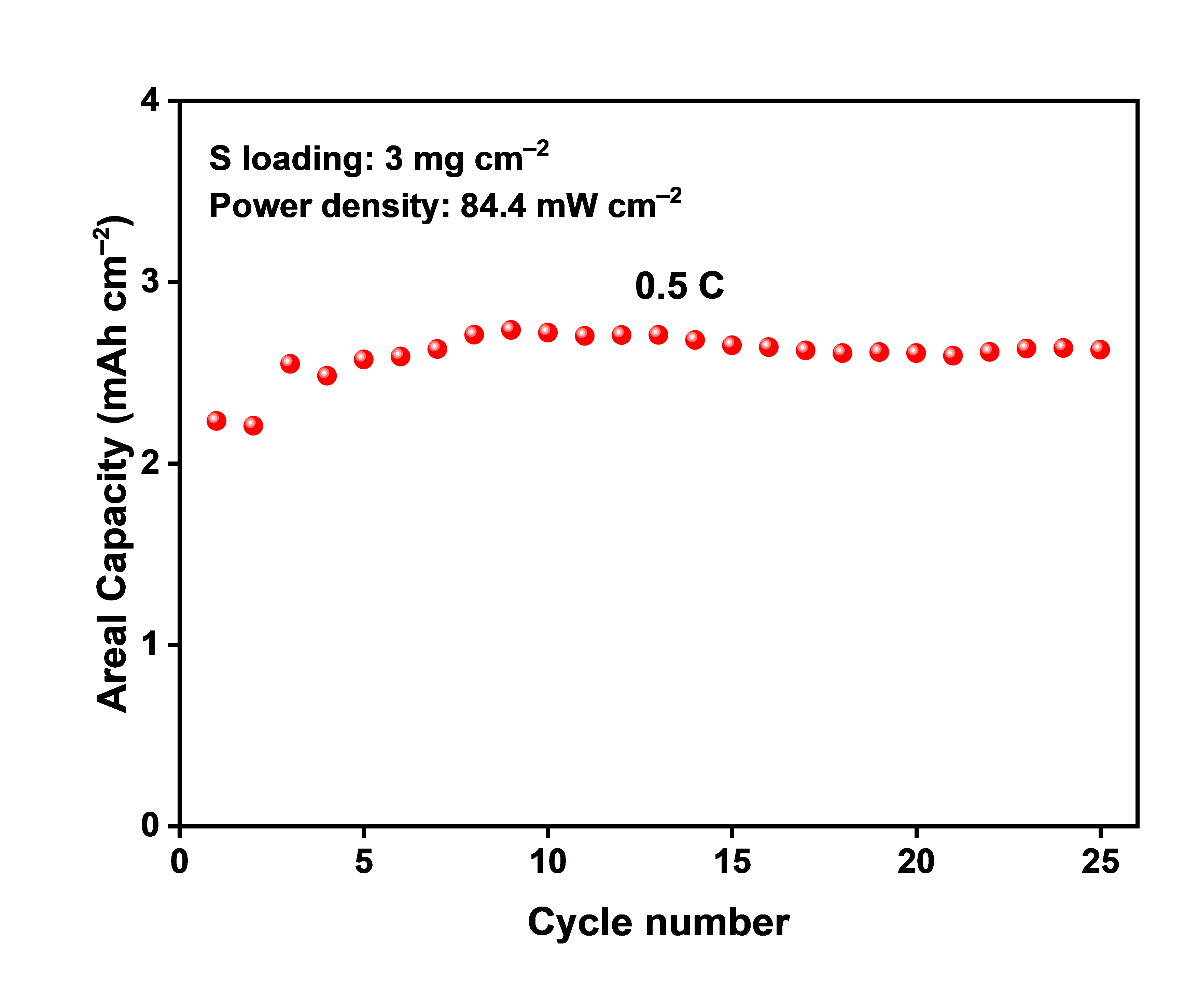


**Fig. S31** Cycling performance with a sulfur loading of 3 mg cm^–2^ at 0.5 C


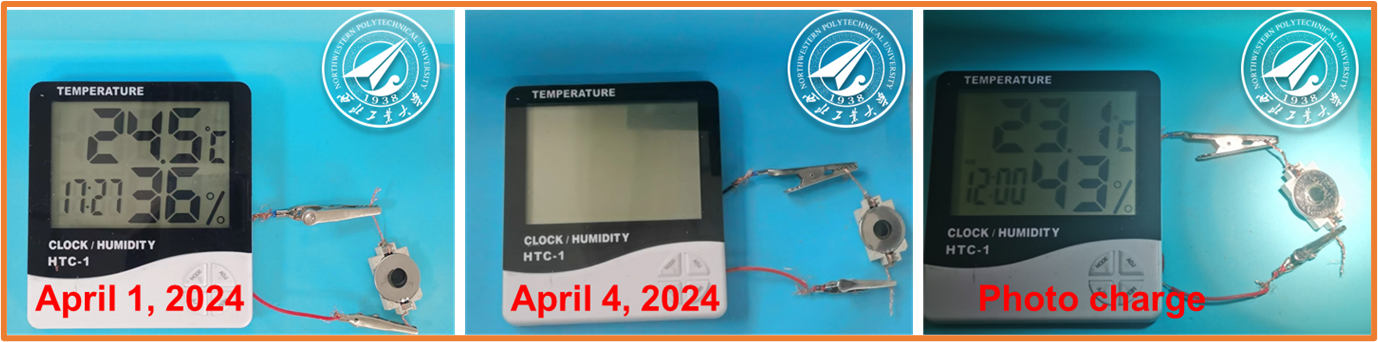


**Fig. S32** Evaluation of the practicability of PPy@N-TiO_2_/CC assembled PALSB: in series with an electronic thermometer

**Tabel S1** The fitted results from EIS analysis

| Cathode | Test condition | R_1_ (ohm) | R_2_ (ohm) | R_3_(ohm) |
| --- | --- | --- | --- | --- |
| PPy@N-TiO_2_/CC | Dark | 10.8 | 71.0 | 23.1 |
|  | Thermal | 10.7 | 61.6 | 21.7 |
|  | Light | 6.4 | 12.5 | 11.4 |
| N-TiO_2_/CC | Light | 8.37 | 19.9 | 13.8 |
| TiO_2_/CC | Light | 9.3 | 18.5 | 26.2 |
